# Leveraging Molecular Mechanics With the uESE Continuum Solvation Model for Efficient Solvation Free Energy Prediction: Impact of Conformation and Extensive Validation

**DOI:** 10.1002/jcc.70252

**Published:** 2025-10-22

**Authors:** Andrew S. Paluch, Jeffrey G. Ethier, Vikas Varshney

**Affiliations:** ^1^ Department of Chemical, Paper, and Biomedical Engineering Miami University Oxford Ohio USA; ^2^ Materials and Manufacturing Directorate Air Force Research Laboratory, Wright‐Patterson Air Force Base Dayton Ohio USA

**Keywords:** conformation, continuum solvation model, electronic structure calculations, molecular mechanics, solvation free energy

## Abstract

The solvation free energy is a fundamental property of a solute directly related to solubility, which in turn is critical for processes ranging from pharmaceutical to materials manufacturing. We seek to develop efficient strategies to predict the solvation free energy, knowing only molecular structure, using electronic structure calculations with the uESE continuum solvation model. Benchmarking on the Minnesota Solvation Database, single conformations generated using the molecular mechanics force field MMFF94 yielded predictive accuracy comparable to reference gas‐phase optimized geometries obtained with electronic structure calculations. Surprisingly, exploring multiple conformations did not consistently improve predictions, suggesting uESE performs well with a single representative input. Evaluation on the independent dGsolvDB1 dataset demonstrated reasonable predictive ability with single molecular mechanics‐generated conformations and some generalizability to novel chemical space. Our findings indicate that combining fast molecular mechanics‐based structure generation with uESE offers a promising approach for efficient and reasonably accurate solvation free energy predictions, with accuracy comparable to or exceeding that of far more computationally intensive methods like explicit solvent molecular dynamics, supporting its utility in high‐throughput screening and machine learning for solubility prediction.

## Graphical abstract

A computationally efficient method is validated for predicting solvation free energies. Using molecular mechanics geometries with the uESE model, our approach provides accurate, high‐throughput data ideal for physics‐informed machine learning and other computational chemistry applications.

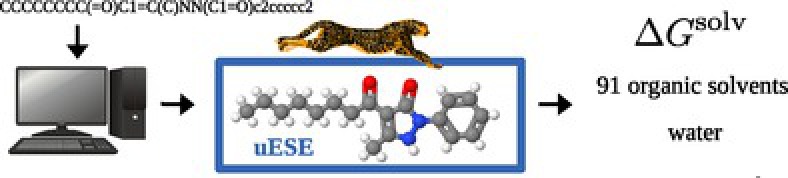

## 1 Introduction

Knowledge, interpretation, and the ability to control the solubility of chemical species are crucial for a wide range of chemical manufacturing processes. For example, the synthesis of pharmaceuticals is commonly carried out in solution, and then subsequently the product needs to be separated, purified, and formulated for delivery. The solubility criteria for each of these steps are likely different, complicating design, with solvents accounting for 80%–90% of the utilized material in a conventional manufacturing process [1, 2, 3]. The manufacturing of polymers is similar, where the solubility is central during synthesis/polymerization, processing, purification, and self‐assembly. For example, a suitable solvent in which the polymer is soluble must be identified for solution polymerization, while a poor solvent (anti‐solvent) may be needed later in the process to facilitate the precipitation of the polymer resin (product) from solution. Similarly, in the recycling of multi‐layer plastic films, the use of selective dissolution requires the ability to identify solvents to target specific polymers in each stage of the process [4].

Quantitative structure activity relationships (QSAR), or quantitative structure property relationships (QSPR) have long been used to predict a range of physical properties, including solubility [5, 6, 7, 8, 9, 10, 11, 12, 13]. Within QSAR, one develops a mapping from a molecular descriptor space to the property of interest. The descriptors are commonly based on the molecular structure and adopted from a database, and may include additional physical properties. More recently, machine learning (ML) has been used, wherein a computer will “learn” and use a specified strategy (i.e., artificial and deep neural networks, random forests, support vector machines, etc.) to develop an optimized functional mapping. However, the predictive power and the ability to extrapolate to novel molecules heavily depend on the quality and relevance of the chosen molecular descriptors and ML architecture.

Here, we are interested in the development of physics‐informed (or chemistry‐/theoretically‐informed) machine learning models [14, 15, 16, 17]. Within this sub‐field, physically relevant properties are incorporated as descriptors during model development. The integration of such physically meaningful features can significantly enhance the interpretability, transferability, and extrapolation capabilities of ML models, particularly crucial for addressing novel chemical spaces in drug discovery and materials science. In the present study, we are specifically interested in the high‐throughput prediction of the solvation free energy as a potential descriptor in the development of models to predict solubility. QSAR methods exist for the prediction of solvation free energy [13], and first principles predictions may be made using molecular simulation [18] and electronic structure calculations in a continuum solvent [19, 20, 21, 22, 23, 24, 25]. Fundamentally, the solvation free energy corresponds to the transfer free energy of a solute molecule from a non‐interacting ideal gas to solution, and is a direct measure of solute‐solvent intermolecular interactions. More importantly, the solvation free energy may be directly related to limiting (or infinite dilution) activity coefficients, Henry's constants, and partition coefficients, and may be used directly to predict phase equilibrium, including solubility [26, 27, 28, 29, 30].

In the present study, we focus on the use of electronic structure calculations in a continuum solvent to predict the solvation free energy. The ability to make first principles predictions in a range of solvents, knowing only the structure of the solute, is appealing for high‐throughput prediction of the solvation free energy as a potential descriptor. However, a limitation of this is the dependence of the input structure on the predicted solvation free energy. During the parameterization of continuum solvation models—including the uESE (universal Easy Solvation Estimation) model [19, 20, 21], the SM*x* family of universal solvation models (e.g., SM12, SM8 and SMD) [22, 23, 24], and the SCCS (Self‐Consistent Continuum Solvation) model [25]—all of the solvation free energies were computed using *rigid*, gas‐phase optimized geometries of the solute. The methods were all parameterized using reference data consisting primarily of “small” molecules, for which conformational changes of the solute upon solvation in solution were expected to be minor. Moreover, it is assumed that conformational changes are implicitly included in the model, which maps from properties computed using the gas‐phase optimized geometry to the experimental solvation free energy. Therefore, the inherent assumption in such approaches is that the single, gas‐phase optimized geometry adequately represents the solute's relevant conformations in solution, or that the impact of conformational flexibility is implicitly captured within the model's parameterization.

However, this assumption may not hold, particularly for larger, multi‐functional solutes. Several cases have been documented in the literature in which the solvation free energy is sensitive to conformational changes of the solute upon solvation. For example, ethane‐1,2‐diol (CAS: 107‐21‐1, HO‐
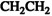
‐OH) contains two terminal hydroxyl groups that are capable of forming intra‐ and inter‐molecular hydrogen bonds. In the gas phase, the dominant conformation involves the formation of intramolecular hydrogen bonds. On the other hand, with a solvent capable of donating and/or accepting hydrogen bonds, in solution there is a competition between intra‐ and inter‐molecular hydrogen bonding [31, 32]. A similar case was documented by Mobley and co‐workers for solutes containing carboxylic acid groups (‐COOH). Considering the case of ibuprofen (CAS: 15687‐27‐1), in the gas phase, the preferred orientation of the carboxylic acid group allows intramolecular hydrogen bonding. However, in solution with a solvent capable of donating and/or accepting hydrogen bonds, we again find that a competition between intra‐ and inter‐molecular hydrogen bonding exists. Furthermore, using molecular simulation, Mobley and co‐workers showed that the quantitative effect on the computed solvation free energy was significant [33, 34].

Within the context of the SM*x* family of continuum solvation models with electronic structure calculations, the issue has come up in the SAMPL (Statistical Assessment of Modeling of Proteins and Ligands) physical property prediction challenges when predicting hydration free energies (i.e., solvation free energies in water) and octanol/water partition coefficients of small drug‐like molecules. Within the SAMPL6 challenge, Jones and Brooks [35] demonstrated that with the SMD solvation model, the use of optimized solution‐phase geometries improved predictions of the octanol/water partition coefficient (solvation free energy in water relative to 1‐octanol) for the 11 solutes considered that resemble fragments of small molecule protein kinase inhibitors. In the SAMPL7 challenge, Rodriguez et al. [36] predicted solvation free energies in water and 1‐octanol for 22 small drug‐like molecules. They compared the case of using only the gas‐phase optimized geometry of the solute with that of using the gas‐phase and solution‐phase optimized geometries to compute the free energy of the gas and solution phases, respectively, which in turn were used to compute the solvation free energy. Using the SM8 solvation model, it was shown that the solvation free energy decreased on average by 2.67 and 1.93 kcal/mol in water and 1‐octanol, respectively, with the use of optimized geometries in the solution phase. Using the SMD solvation model, the solvation free energy decreased by 1.45 and 0.87 kcal/mol in water and 1‐octanol, respectively. To put the change in context, during the original parameterization of the SMD solvation model, the developers reported a mean unsigned error of 0.59 kcal/mol for 274 neutral solute molecules in water, with the theory/basis set with the smallest error [23]. Furthermore, in the work of Rodriguez et al. [36] the change in the geometry of the solute in solution relative to the gas phase was relatively minor. In solution, a local geometry optimization was performed by starting with the gas‐phase optimized structure of the solute; alternative conformations were not considered. It may be possible that the change would be even larger if additional conformations were considered.

An earlier study by the SM*x* developers also noted the sensitivity of the computed solvation free energy with subtle changes in geometry [37]. In that work, the authors performed calculations of the hydration free energy of 17 small molecules as part of an informal blind challenge [38]. The authors used two different theories and basis set combinations to obtain the optimized gas‐phase geometry of each solute, which was subsequently used to compute the hydration free energy with the SM8 continuum solvent model using the same theory and basis set combination [37]. With this subtle change in geometry, the root mean squared error in the predictions decreased from 1.25 to 1.14 kcal/mol. The authors then found that if optimization of the geometry of the solute was performed in the SM8 model for water (i.e., in solution), the root mean squared error further decreased from 1.14 to 1.08 kcal/mol. This reduction in error resulting from even minor variations in the input geometry underscores the inherent sensitivity of continuum solvation models to the precise conformation of the solute.

While earlier works have demonstrated that the predicted solvation free energy is sensitive to the solute geometry when using electronic structure calculations with continuum solvents, the studies were limited to on the order of 10 unique solute molecules studied as discussed earlier. In the present study, we investigate the effect of solute conformation on the solvation free energy over a large set of reference systems. All solvation free energy calculations were performed using the recently developed uESE model [19, 20, 21]. While continuum solvation models can be paired with various charge models, the uESE model was developed using CM5 partial atomic charges [19, 39]. The claimed advantages of CM5 charges over standard methods include: (1) predicted dipole moments are in general more accurate, (2) the charges are essentially independent of the basis set used, (3) the charges exhibit a weaker dependence on the molecule conformation, and (4) the model performance does not suffer from ill conditioning for buried atoms in larger molecules [39]. Given these advantageous properties of the CM5 charge model, including its reduced sensitivity to molecular conformation and basis set, it is plausible that the uESE model may exhibit a lower susceptibility to variations in computed solvation free energies arising from different solute conformations and the level of electronic structure theory employed compared to solvation models employing other charge schemes.

We note that the most recent SM*x* solvation model, SM12, can also make use of CM5 charges [24]. However, the practical application of SM12 is currently somewhat limited by the less widespread availability of compatible electronic structure calculation software. In contrast, the uESE model is parameterized for the same 92 solvents, making use of the same reference training database, with the ability to extend to additional solvents [19]. Calculations with uESE require only the solute molecular geometry (Cartesian coordinates) and the corresponding CM5 partial atomic charges. The CM5 partial atomic charges may be computed from a single‐point energy calculation (in vacuum) with suitable electronic structure calculation software. The solvation free energy may then be computed using software made freely available by the developers [40].

The primary goal of this manuscript is to thoroughly assess the viability of using the uESE continuum solvation model for efficient, high‐throughput predictions of solvation free energies. Given uESE's reliance on CM5 partial atomic charges, which exhibit a reduced sensitivity to molecular geometry and basis set effects, we explore the potential of employing computationally inexpensive molecular mechanics to generate solute conformations. This includes a detailed comparison of using single low‐energy conformations versus considering multiple conformations to achieve a balance between accuracy and computational efficiency.

Here, we first assess the performance of the uESE model to predict the solvation free energy of neutral solute molecules in the Minnesota Solvation Database version 2012 [41]. The Minnesota Solvation Database was used to parameterize the uESE and SM*x* solvation models. We compare the use of reference gas‐phase optimized geometries provided in the Minnesota Solvation Database to geometries efficiently generated using molecular mechanics with the MMFF94 force field [42, 43, 44, 45, 46, 47]. Using molecular mechanics, we further compare the use of a single low‐energy conformation to the use of multiple conformations. We find that the use of a single low‐energy conformation efficiently generated using molecular mechanics outperforms the use of multiple conformations, and is comparable to the use of the reference geometries. We subsequently assess the performance of the uESE model with a single conformation on an independent subset of the dGsolvDB1 database with the Minnesota Solvation Database removed [13]. The geometries generated using molecular mechanics can readily be performed on a desktop computer and are readily amenable to high‐throughput calculations.

Ultimately, our comprehensive benchmarking demonstrates that uESE delivers efficient and accurate solvation free energy predictions that are competitive with, and often surpass the performance of, more computationally demanding explicit solvent methods. This positions uESE as a robust and promising approach for high‐throughput computational studies and machine learning applications in solubility prediction.

## 2 Computational Details

**FIGURE 1:**
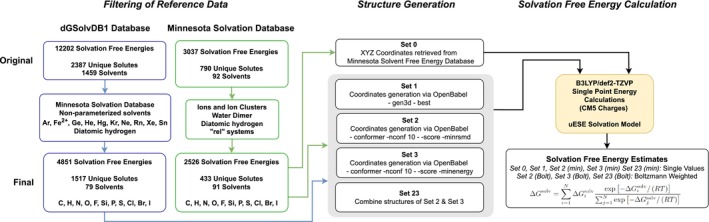
Flowsheet summarizing the reference database filtering and calculations performed as detailed in the “Computational Details” section.

A summary of the computational details and database filtering to be described in detail next may be found in Figure 1. All calculations of the solvation free energy, $\Delta G^{\text{solv}}$, were performed with the recently developed uESE (universal Easy Solvation Estimation) model [19, 20, 21], using software made freely available by the developers [40]. For each solute, solvation free energy calculations were performed in all 92 solvents for which uESE is parameterized. Calculating the solvation free energy for each solute with uESE requires as input the solute geometry (Cartesian coordinates) and CM5 partial charges [39]. In all cases, for a given geometry, CM5 partial charges were obtained from a single point energy calculation using the electronic structure calculation software Gaussian 16 Revision C.01 [48] using the B3LYP/def2‐TZVP theory/basis set [49, 50]. This is the same theory/basis set combination used in the parameterization of uESE. The choice of CM5 partial atomic charges [39] is integral to the uESE model. As highlighted by the original developers of uESE [19] and its predecessor, xESE [20], CM5 charges were specifically selected for their robust performance in estimating solvation free energies. This robustness stems from CM5's derivation from Hirshfeld population analysis, which yields charges that are notably less sensitive to basis set size or choice compared to charges derived from Löwdin analysis or even electrostatic potentials (ESP) like CHELPG or Merz–Kollman [24, 39]. While ESP‐derived charges are widely used, particularly in classical force fields for molecular simulations (e.g., RESP and CHELPG), CM5 charges offer distinct advantages that align with the philosophy of continuum solvation models like uESE. Specifically, CM5 charges are known for their transferability, are less computationally expensive to obtain than ESP charges, and, crucially for this study, exhibit a weaker dependence on molecular conformation [24, 39]. They also avoid issues of ill conditioning for buried atoms in larger molecules that can sometimes affect ESP‐derived charges. Indeed, the utility of CM5 charges extends beyond implicit solvation, with pioneering work demonstrating their effectiveness, when appropriately scaled, for accurate hydration free energy predictions using explicit solvent molecular simulations with popular force fields like OPLS‐AA [51]. More recent work has further supported the use of CM5‐based partial charges for condensed‐phase properties [52]. Moreover, a key practical advantage is that CM5 charges are readily available from various quantum chemical programs, including Gaussian, making their integration into high‐throughput computational workflows straightforward. Therefore, the use of CM5 charges is a fundamental and justified aspect of the uESE methodology rather than an arbitrary choice.

With respect to solute geometry, four sets of calculations were performed. In all cases, geometries were generated by molecular mechanics with the software Open Babel 3.0.0 [53, 54], using the Merck Molecular Force Field (MMFF94; –ff mmff94) [42, 43, 44, 45, 46, 47]. MMFF94 was selected over other popular force fields like GAFF or OPLS‐AA for several key reasons. Foremost, our datasets include Si‐containing molecules, and MMFF94 is uniquely capable of modeling these, unlike GAFF, which is not parameterized for silicon atoms [55]. This ensured broad applicability across the diverse chemical space of our databases. Furthermore, while OPLS‐AA is a prominent force field, it is not implemented for geometry generation within Open Babel, the software employed in this study for efficient, high‐throughput conformation generation [56].

It is crucial to clarify that MMFF94 was employed solely for efficient *intramolecular* geometry optimization and conformational energy ranking in the gas phase. This involves generating and cleaning up initial structures and guiding the conformer search towards reasonable geometries [56]. MMFF94 is the default force field used by Open Babel's robust gen3d and conformer generation functionalities, recognized for producing high‐quality input structures efficiently [56]. The force field does not model any intermolecular interactions (solute‐solvent, solute‐solute, or solvent‐solvent) within our workflow. Instead, the MMFF94‐generated geometries are used as input for subsequent electronic structure calculations to derive CM5 partial atomic charges, which, along with the geometry, are then fed into the uESE continuum solvation model for the final solvation free energy computation. As previously discussed in the context of CM5 charges, their inherent weaker dependence on molecular conformation further suggests that the precise choice of force field for geometry generation has a reduced impact on the final solvation free energy predictions. In this context, MMFF94 is well‐suited for its specific role.

In the first set of calculations (set 1), we used the structure of a single conformation for each solute generated using the flags “–gen3d –best”, corresponding to the use of geometry optimization and conformer searching to predict the structure of the lowest energy conformer [56, 57]. For each solute, the structure in set 1 is used as the starting point for sets 2 and 3, which will be described next.

In the second set of calculations (set 2), for each solute we use the structure of set 1 to generate 10 conformations using the flags “–conformer –nconf 10 –score minrmsd –writeconformers”. This uses a genetic algorithm to generate up to 10 conformations optimized for RMSD (root mean squared deviation) diversity with geometry optimization (or energy minimization) of the conformers used during the search process. The use of geometry optimization ensures that the proposed conformers are reasonable and avoid steric clashes. The number of possible conformations is dependent on the number of torsional angles within the molecule, and here we set the maximum number of conformations to use at 10, although additional conformations may be possible. Likewise, fewer than 10 unique conformations may be found. With RMSD diversity, the goal is to sample different regions of the conformational space. We then perform a final geometry optimization (or energy minimization) of the final conformer candidates with the flag “–minimize”.

Similar to set 2, for the third set of calculations (set 3), for each solute we use the structure of 1 to 10 conformations generated using the flags “–conformer –nconf 10 –score minenergy –writeconformers”. This differs from set 2 in that a genetic algorithm is used to generate up to 10 of the lowest‐energy conformers with geometry optimization of the conformers used during the search process. We again perform a final geometry optimization (or energy minimization) of the final conformer candidates with the flag “–minimize”.

Finally, in the fourth set of calculations (set 23), for each solute, we combine the structures of sets 2 (RMSD diversity of conformers) and 3 (lowest energy conformers).

For each solute structure, we perform an independent single‐point energy calculation using the electronic structure calculation software Gaussian 16 to compute CM5 partial charges, which are subsequently used to compute the solvation free energy using the uESE model. For sets 2, 3, and 23, for a given solute, we may have more than one conformation, with a value of the solvation free energy in each solvent for each conformation. We employed two schemes to determine the reported value of the solvation free energy for each solute in each solvent. In the first case, for each solute within each solvent, we select the minimum (“min”) value of the solvation free energy. In the second case, for each solute within each solvent, we compute the Boltzmann (“Bolt”) average value of the solvation free energy as:

(1) $$\Delta G^{\text{solv}}=\overset{N}{\underset{i=1}{\sum }}\Delta G_{i}^{\text{solv}}\frac{\exp\left[-\Delta G_{i}^{\text{solv}}/\left(RT\right)\right]}{\sum_{j=1}^{N}\exp\left[-\Delta G_{j}^{\text{solv}}/\left(RT\right)\right]}$$

where the summations are both over all *N* conformations, R is the molar gas constant, T=298 K is the absolute temperature, and $\Delta G_{i}^{\text{solv}}$ and $\Delta G_{j}^{\text{solv}}$ corresponds to the solvation free energy of conformation i and j, respectively.

### 2.1 Minnesota Solvation Database

The effect of solute conformation on the computed solvation free energy ($\Delta G^{\text{solv}}$) was assessed using the Minnesota Solvation Database version 2012 [41, 58]. The Minnesota Solvation Database contains 3037 experimental solvation free energies for 790 unique solutes (neutrals and ions) in 92 solvents, composed of H, C, N, O, F, Si, P, S, Cl, Br, and I. From this dataset, we first eliminated all solvation free energies wherein the solute was an ion or cluster of ions. We additionally eliminated the case where the solute was a water dimer (one system, water in water) and diatomic hydrogen (three systems). Here, our focus is on assessing the effect of the conformation on neutral solutes, where the conformation of the solute can quickly be generated using molecular mechanics; MMFF94 is unable to model diatomic hydrogen. This resulted in a reduced set of 2670 solvation free energies.

We next eliminated the 144 systems tagged as “rel” (relative), which correspond to solvation free energies in an organic phase derived from partitioning data (or the transfer free energy) from water to the organic phase. The majority of these systems (103) are derived from octanol‐water partitioning.^1^ The final data set used contained 2526 solvation free energies for 433 unique solutes in 91 solvents. Data was available in all parameterized solvents except methanol.

It should be noted that the Minnesota Solvation Database was used to parameterize the uESE model [19]. In that work, the developers excluded only solvation free energies derived from partitioning data. Likewise, the SM*x* family of universal solvation models (e.g., SM12, SM8, and SMD) was parameterized using a subset of the Minnesota Solvation Database [22, 23, 24].

The SM*x* family of universal solvation models and uESE were parameterized using rigid, gas‐phase optimized geometries of the solute. The Minnesota Solvation Database version 2012 additionally contains the gas‐phase optimized molecular geometries, obtained using electronic structure calculations, in Cartesian coordinates for the corresponding solute. These molecular geometries were used in the parameterization of SM12, while SMD and SM8 used gas‐phase optimized geometries obtained using a different theory and a smaller basis set [24]. While the earlier ESE model optimized for hydration free energies made use of the molecular geometries from the Minnesota Solvation Database [21], it is not clear if molecular geometries from the Minnesota Solvation Database were adopted during the parameterization of uESE [19].

In the present study, for the Minnesota Solvation Database, we have performed an additional set of calculations, which we term set 0. For this set, the calculations were the same as described earlier, only here we used the reference gas‐phase optimized geometries from the Minnesota Solvation Database. We additionally make comparison to uESE and SMD predictions using the same B3LYP/def2‐TZVP theory/basis set provided in the Supporting Information of the original uESE (included SMD with organic solvents) and xESE (included SMD with water) publications [19, 20]. We refer to this set of reference calculations as “ref”.

### 2.2 dGsolvDB1 Database

The Minnesota Solvation Database was used for the parameterization of uESE. We therefore subsequently performed calculations for an independent subset of the dGsolvDB1 database [13]. The original dGsolvDB1 database contains experimental solvation free energies from the Minnesota Solvation Database, along with the published CompSol [63] and FreeSolv [18] databases, and values from the published works of Abraham, Acree and co‐workers [64, 65, 66, 67, 68, 69, 70, 71, 72, 73, 74, 75, 76, 77, 78, 79, 80, 81, 82, 83, 84, 85, 86, 87, 88, 89, 90, 91, 92, 93, 94, 95]. During the development of the dGsolvDB1 database, when CompSol had multiple values for a system, the dGsolvDB1 authors report the average which we use in this work. The original dGsolvDB1 database contained 12,202 experimental solvation free energies for 2387 unique solutes at 298 K. From this, data from the Minnesota Solvation Database was removed. The Minnesota Solvation Database is not open‐source and was therefore not included in the published version of the dGsolvDB1 database by the authors. We then eliminated cases involving solvents for which uESE is not parameterized and solutes unable to be modeled using MMFF94.^2^ This resulted in a subset of the dGsolvDB1 database, independent of the Minnesota Solvation Database, containing 4851 experimental solvation free energies for 1517 unique solutes and 79 solvents.

The dGsolvDB1 database did not provide solute or solvent names, but instead provided SMILES and InChI strings. We made use of the SMILES for all of the filtering and solute structure generation. After performing our initial set of calculations with uESE, we observed large deviations for dibutyl phosphate (CAS: 107‐66‐4) and dioctyl phosphate (CAS: 3115‐39‐7) in water. We found the SMILES provided in the dGsolvDB1 database corresponded to the deprotonated form with a net charge of −1. While we expect the deprotonated form to be the dominant form in water at near neutral pH, we trust the original source data corresponds to the neutral form, consistent with the rest of the database [96]. We therefore updated the SMILES of dibutyl phosphate and dioctyl phosphate to be that of the neutral form. Checking, no additional non‐neutral solutes were found.

## 3 Results and Discussion

Throughout this section, the number of torsional angles (or rotors) not involving hydrogen atoms is used as a measure of the solute's conformational flexibility. To facilitate discussion, the use of “torsional angle” will imply the exclusion of hydrogen atoms.^3^ The number of torsional angles not involving hydrogen atoms and other descriptors was obtained directly from Open Babel 3.0.0 using the flag –append “atoms bonds rotors HBD HBA1 HBA2” to append the frequency of occurrence of each descriptor to the provided molecular structure [20].

### 3.1 Minnesota Solvation Database

**FIGURE 2:**
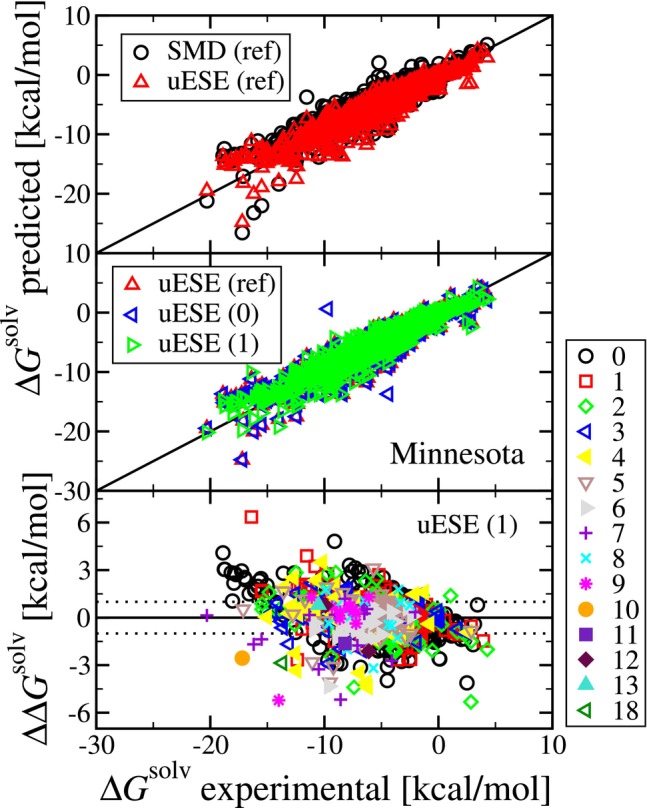
The top two panes contain parity plots of the predicted versus experimental solvation free energy ($\Delta G^{\text{solv}}$) from the Minnesota Solvation Database. The top pane contains the reference (“ref”) uESE and SMD predictions from the Supporting Information of References [19, 20] and [21]. The middle pane contains the reference uESE predictions along with the uESE predictions from this work for sets 0 and 1. The bottom pane is a residual plot of the predicted relative to the experimental solvation free energy ($\Delta \Delta G^{\text{solv}}$) for set 1 versus the experimental solvation free energy. The symbol shape and color correspond to the number of torsional angles. The dotted lines illustrate a range of ±1 kcal/mol and are drawn as a reference.

Let us first consider the use of a single conformation in the calculation of solvation free energy ($\Delta G^{\text{solv}}$). The results are summarized in Figure 2, and the detailed results are also tabulated in spreadsheets in Supporting Information 2 (SI‐2) accompanying the electronic version of this manuscript.

In the upper panel of Figure 2, we first compare the reference (“ref”) SMD and uESE predictions performed by the uESE developers [19]. With SMD, where the solvent is water (N=388) and non‐water (N=2138), we have a mean unsigned error (MUE) of 1.15 and 0.73 kcal/mol, respectively.^4^ With uESE, the overall error is smaller. Where the solvent is water and non‐water, we have a MUE of 0.99 and 0.50 kcal/mol, respectively.

In the middle panel, we compare the reference uESE predictions with those made here using a single conformation. Our set 0 predictions made use of the rigid gas‐phase optimized geometries provided as part of the Minnesota Solvation Database. We find that the set 0 and reference predictions are in very close agreement, except for two outliers. With set 0, where the solvent is water and non‐water, we have a MUE of 1.03 and 0.50 kcal/mol, respectively. For both of the outliers, water is the solvent. The first system corresponds to the herbicide bromacil (CAS: 314‐40‐9), where the uESE set 0 prediction is 0.625 kcal/mol and the reference hydration free energy is −9.7 kcal/mol. While the prediction here indicates less favorable solvation, the reference uESE prediction is −13.573 kcal/mol, which indicates more favorable solvation. The second system corresponds to the insecticide phorate (CAS: 298‐02‐2). Here, the uESE set 0 prediction is −13.698 kcal/mol, and the reference hydration free energy is −4.4 kcal/mol. On the other hand, the reference uESE prediction is in close agreement and is −4.812 kcal/mol. The cause of the discrepancy is unclear. As noted earlier, while the earlier ESE model optimized for hydration free energies made use of the molecular geometries from the Minnesota Solvation Database [19], it is not explicitly stated whether or not the molecular geometries of the Minnesota Solvation Database were adopted during the parameterization of uESE [36]. We also repeated the calculations with Gaussian 16 Revision A.03 as used in the parameterization of uESE, compared to Revision C.01 used in the present study [39], but the results were unchanged. All other predictions were in excellent agreement. There were only 4 other systems for which the absolute deviation was greater than 0.1 kcal/mol, and only one of these systems had a deviation greater than 0.16 kcal/mol (0.873 kcal/mol, octafluoropropane in water).

In the middle panel, we additionally compare the reference uESE predictions to our set 1, where we also make use of a single conformation, here obtained using Open Babel using molecular mechanics with MMFF94. With set 1, where the solvent is water and non‐water, we obtain a MUE of 0.91 and 0.51 kcal/mol, respectively. Interestingly, the MUE in water is slightly less than for the set of reference predictions, whereas the MUE in non‐water solvents is very slightly higher. Nevertheless, we find that the results obtained using a single conformation generated using molecular mechanics (set 1) are in close agreement with the reference set. Moreover, compared to set 0, the two large outliers were not observed. Investigating the outliers further, uESE computes the total solvation free energy, $\Delta G^{\text{solv}}$, as a sum of an electrostatic (COSMO) contribution resulting from solute–solvent intermolecular electrostatic interactions and a second “correction” term that accounts for cavity formation and dispersion and repulsion interactions [19, 20]. Comparing sets 0 and 1 for bromacil, the electrostatic contribution is −9.469 and −9.556 kcal/mol, respectively. Given the low conformational dependence of CM5 partial atomic charges, we see only a minute change in the electrostatic contribution. On the other hand, for sets 0 and 1, the “correction” term is 0.625 and −10.513 kcal/mol, respectively. For set 0, the solvent‐independent total cavity (van der Waals) area and volume computed during the uESE calculation are 237.145 Å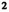 and 192.207 Å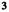, respectively, as compared to the set 1 values of 241.242 Å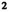 and 193.243 Å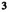, respectively. The result highlights that subtle changes in geometry can have a large impact on the resulting $\Delta G^{\text{solv}}$. The results are similar for the second outlier (i.e., phorate).

Finally, the bottom panel is a residual plot of the predicted $\Delta G^{\text{solv}}$ of our set 1 relative to the experimental value, versus the experimental value, where the symbol corresponds to the number of torsional angles. This will be explored more shortly, but there is no apparent trend in the observed error with the number of torsional angles. We expect that this is the result of the parameterization of uESE, in which a single geometry was used during the parameterization.

**FIGURE 3:**
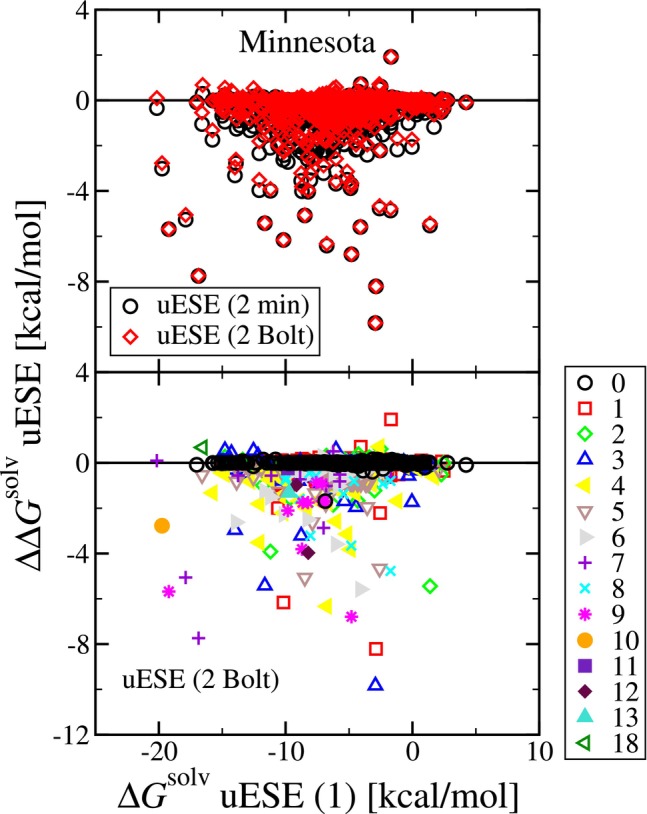
The top pane contains a residual plot of set 2 (multiple conformations, RMSD diversity) predicted solvation free energy relative to set 1 (single conformation), versus set 1. Within the set 2 predictions results are shown for the use of the minimum (“min”) $\Delta G^{\text{solv}}$ and the Boltzmann weighted (“Bolt”) value. The bottom pane is a residual plot of the set 2 Bolt $\Delta G^{\text{solv}}$ relative to the set 1 value, versus the set 1 value. The symbol shape and color correspond to the number of torsional angles.

Next, in Figure 3, we examine the effect of including multiple conformations in the calculation of $\Delta G^{\text{solv}}$. In this analysis, we consider set 2 (1 to 10 conformations based on RMSD diversity), using the minimum (“min”) $\Delta G^{\text{solv}}$ and the Boltzmann weighted (“Bolt”) value. The top panel is a residual plot of the set 2 predicted $\Delta G^{\text{solv}}$ relative to set 1, versus the set 1 $\Delta G^{\text{solv}}$. First, we observe that overall, the difference in using the minimum $\Delta G^{\text{solv}}$ among the conformations is minor as compared to using the Boltzmann weighted value of $\Delta G^{\text{solv}}$. Second, we observe that the effect of considering multiple conformations is significant as compared to using just a single conformation. Third, in general, the effect of considering multiple conformations is to decrease the predicted $\Delta G^{\text{solv}}$, corresponding to more favorable solvation. Relative to set 1, the average change in the Boltzmann weighted values was −0.186 kcal/mol, and the average absolute change was 0.205 kcal/mol. This result is similar to the findings of Rodriguez et al. [96]. The solute geometry in set 1 corresponds to the gas‐phase optimized geometry. The optimized geometry in solution is likely different and would result in a decrease in the calculated value of $\Delta G^{\text{solv}}$. While the solvent is not considered in the generation of conformations, the goal of generating a set of conformations is to obtain a structure or structures representative of the solution phase.

In the bottom panel, we plot the same data for the Boltzmann weighted values, where we vary the symbol color and shape according to the number of solute torsional angles. We find that for the case of 0 torsional angles, in general, the change is not significant. However, the change is, in general, noticeable when we have at least one torsional angle. For the case of 0 torsional angles, the average value of the set 2 Bolt $\Delta G^{\text{solv}}$ relative to the set 1 value is −0.002 kcal/mol (N=1176). For the case of at least one torsional angle, the average value of the set 2 Bolt $\Delta G^{\text{solv}}$ relative to the set 1 value is −0.347 kcal/mol (N=1350), with the largest decrease being −9.83 kcal/mol corresponding to pentanal (CAS: 110‐62‐3) in water. Interestingly, pentanal has just three torsional angles, no hydrogen bond donating, and one hydrogen bond accepting site. We therefore find that while in general the effect of considering multiple conformations is to decrease the computed $\Delta G^{\text{solv}}$ for the case of solutes with at least one torsional angle, the impact varies and does not simply scale with the number of torsional angles, and the average decrease is less than the overall MUE and expected error in the calculations. Further, for the case of at least one torsional angles, the average value of the set 1 $\Delta G^{\text{solv}}$ relative to the reference (experimental) value is −0.022 kcal/mol (N=1350), while the average value of the set 2 Bolt $\Delta G^{\text{solv}}$ relative to the reference (experimental) value decreases to −0.368 kcal/mol.

**FIGURE 4:**
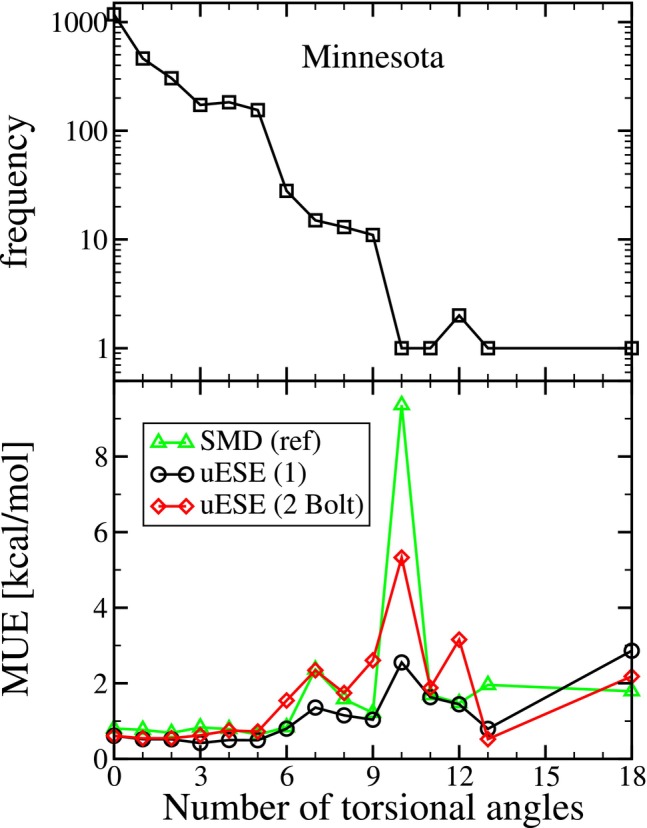
The top pane is the frequency of systems versus the number of torsional angles of the solute. The bottom pane is the mean unsigned error (MUE) versus the number of solute torsional angles. Results are presented for the SMD reference (“ref”) calculations [58], uESE set 1 (single conformation), and uESE set 2 Bolt (multiple conformations, RMSD diversity, Boltzmann weighted) versus reference values from the Minnesota Solvation Database.

The effect of the number of torsional angles on the calculation of $\Delta G^{\text{solv}}$ is further investigated in Figure 4. In the top panel, we plot the number (frequency) of solute‐solvent pairs versus the number of torsional angles. Of the 2526 systems, 1176 have 0 torsional angles, followed by 462 systems with 1 torsional angle. Over half of the systems contain 1 or 0 torsional angles, reiterating that the Minnesota Solvation Database is composed primarily of relatively small molecules. In the middle panel, we plot the mean unsigned error (MUE) of the reference SMD predictions, and our sets 1 and 2 Bolt uESE predictions versus the number of torsional angles. Comparing the reference SMD predictions and our set 1 uESE predictions, we see a large difference for 10 torsional angles where the SMD error jumps to 9.36 kcal/mol. However, it is important to note that this corresponds to just a single system. Nevertheless, the lower and more consistent error observed with uESE could also be related to its use of CM5 atomic charges, which have been found to have a lower conformational dependence [41, 58].

Comparing the MUE of our sets 1 and 2 Bolt uESE predictions, we find that both are in close agreement when we have two or fewer torsional angles. However, for three or more torsional angles, we begin to observe a difference. We find that, in general, the error for set 2 (multiple conformations) is greater than set 1 (a single conformation). This observation is interesting because we expect the use of multiple conformations to result in a better physical representation of our system. Despite this, it results in a larger error. We hypothesize that this is the result of the original parameterization of uESE that used a single, optimized gas‐phase structure.

**FIGURE 5:**
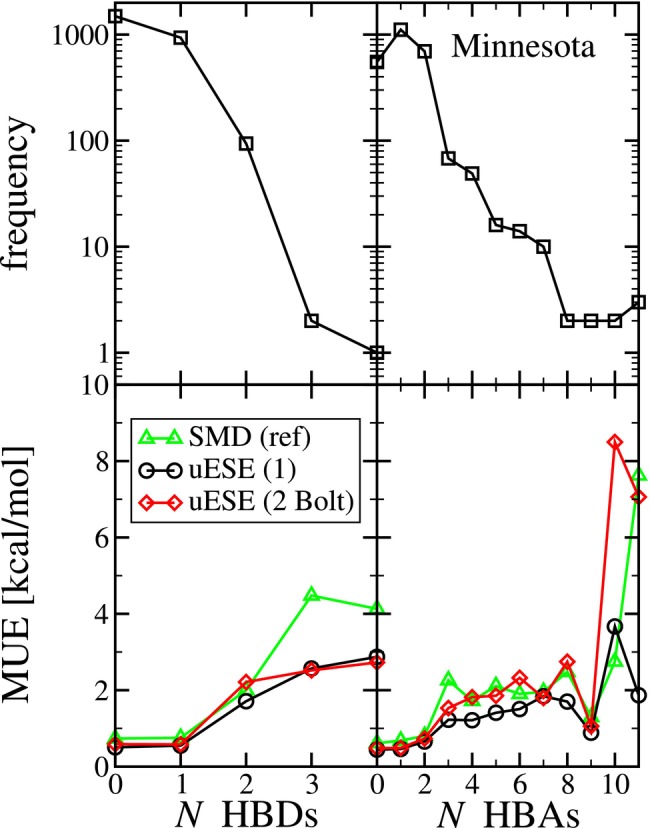
The top row is the frequency of systems versus the number (*N*) of hydrogen bond donor sites (HBDs) and the number (*N*) of hydrogen bond acceptor sites (HBAs) of the solute in columns 1 and 2, respectively. The bottom row is the mean unsigned error (MUE) versus the number (*N*) of hydrogen bond donor sites (HBDs) and the number (*N*) of hydrogen bond acceptor sites (HBAs) of the solute in columns 1 and 2, respectively. The results correspond predicted (uESE set 1, single conformation) versus experimental solvation free energy ($\Delta G^{\text{solv}}$) from the Minnesota Solvation Database.

Similarly, in Figure 5, we investigate the error versus the number of hydrogen bond donors (HBD) and hydrogen bond acceptors (HBA) sites in the solute. Considering first HBD, of the 2526 systems, over half (1493 systems) have a solute with 0 hydrogen bond donor sites, and 936 systems have a solute with 1 hydrogen bond donor site. We again find that overall, the error with uESE appears to be less than SMD. Also, comparing the two sets of uESE predictions, we notice the MUE is noticeably larger for 2 hydrogen bond donor sites for set 2 (2.213 kcal/mol) as compared to set 1 (1.711 kcal/mol). Interestingly, the 94 systems where the solute has 2 hydrogen bond donor sites include the 1 system where the solute has 10 torsional angles. This system corresponds to bensulfuron‐methyl (CAS: 83055‐99‐6). Additionally, for all of the sets of predictions, we find that the MUE increases with respect to the number of hydrogen bond donor sites.

Considering the next HBA, of the 2526 systems, 552 systems have 0 hydrogen bond acceptor sites, 1112 have 1 hydrogen bond acceptor site, and 696 have 2 hydrogen bond acceptor sites. With uESE, the largest error occurs for the systems with 10 hydrogen bond acceptor sites. However, this corresponds to just two systems where water is the solvent. The range of the number of hydrogen bond acceptor sites is much greater than the range of the number of hydrogen bond donor sites. Nonetheless, the error is comparable except for the two outliers of 10 and 11 HBAs. Note that here HBA corresponds to HBA1 in Open Babel [58]; the results for HBA2 are tabulated in SI‐2 accompanying the electronic version of this manuscript and are consistent with HBA1.

Within Supporting Information 1 (SI‐1), a discussion is also provided with respect to the error versus the number of non‐hydrogen (intramolecular) bonds (Figure S1) and atoms (Figure S2) as a measure of the size of the solute, with the results consistent with those presented already. While the focus of this discussion has been on the effect of the conformation on the predicted $\Delta G^{\text{solv}}$ based on various measures of the size of the solute molecules, detailed results are provided in the spreadsheets in SI‐2 accompanying the electronic version of this manuscript. We tabulate all of the predictions and descriptors used to characterize the solute molecules, and a summary of the error broken down by solvent (including non‐water) and uESE solvent class. We additionally provide a summary of the error based on the solute descriptor (torsional angles, atoms and bonds, hydrogen bond donor sites, and hydrogen bond acceptor sites). All of the errors based on the solute descriptor are provided overall, when water is the solvent, and for non‐water solvents. Note, in SI‐2 we have not included the values of the reference solvation free energies from the Minnesota Solvation Database version 2020 due to the license under which the values are published [97]. For academic, government, and other non‐profit organizations, the database is free of charge and can readily be downloaded from the author's website [97]; a license and fee is required for commercial use [98].

Building upon the findings discussed above, the use of a single conformation generated with molecular mechanics proved to be at least comparable to the use of the reference geometry and superior to considering multiple conformations. Furthermore, recalling the claimed advantage of CM5 charges exhibiting a weaker dependence on molecular conformation (as mentioned in the Introduction), our analysis of the dGsolvDB1 database will therefore focus on set 1, which employs a single MMFF94 conformation. This approach aligns with the expectation that uESE, leveraging CM5 charges, may provide reasonable accuracy even with geometries that are not high‐level gas‐phase optimized structures.

#### 3.1.1 Additional Benchmarking

To provide context for the performance of uESE, we compare our results against other notable solvation models. With set 1 (single MMFF94 conformation), for the Minnesota Solvation Database, uESE yielded a mean unsigned error (MUE) of 0.91 kcal/mol for water as a solvent and 0.51 kcal/mol for non‐aqueous solvents, respectively.

For direct comparison with similar implicit solvent approaches that were parameterized using the Minnesota Solvation Database, we first consider SM12. As the most recent SM*x* solvation model, SM12 shares conceptual similarities with uESE (e.g., both employ CM5 charges). Notably, the Minnesota Solvation Database itself was originally developed by the creators of the SM*x* models for the purpose of their parameterization. During its parameterization, SM12 achieved an MUE of 0.55 kcal/mol for neutral solutes in non‐aqueous solvents (N = 2129, using CM5 charges computed with M06‐2X/6‐31G*) and 0.76 kcal/mol for solvation in water (N = 374). It is important to note that both uESE and SM12 are being evaluated here against a database that was included in their respective parameterizations. Thus, this comparison primarily reflects their ability to reproduce known data within their training set, rather than their true predictive power.

Another recent electronic structure‐based implicit solvation model, openCOSMO‐RS, also reported an MUE of 0.45 kcal/mol for N = 2129 systems from the Minnesota Solvation Database [98] (excluding cases where water was a solute and xylene, treated as an isomeric mixture). Similar to SM12 and uESE, openCOSMO‐RS derives solvation free energies from molecular structure, utilizing a single conformation per solute during both parameterization and prediction. Consistent with our findings and the inherent properties of CM5 charges in uESE, the developers of openCOSMO‐RS additionally noted a weak dependence of their results on the use of multiple conformations [13]. Like SM12 and uESE, openCOSMO‐RS also utilized the Minnesota Solvation Database in its parameterization, making this a comparison of model reproduction capabilities rather than external predictability.

While the MUEs reported for SM12 and openCOSMO‐RS appear slightly lower than those obtained with uESE for the Minnesota Solvation Database, it is challenging to draw definitive conclusions regarding their comparative predictive power in this context. As noted, all three models were parameterized using this database, making these comparisons primarily reflective of their ability to reproduce their respective training data. A more rigorous assessment of their true predictive performance would necessitate comparison against independent, external validation datasets, which warrants further investigation.

In contrast to these implicit solvation models, we also provide a benchmark against a recent explicit solvent molecular simulation approach, which offers a different paradigm for capturing solvation physics. Wang and co‐workers recently developed the ABCG2 charge model for use with the second‐generation GAFF (GAFF2) force field in molecular simulations [19]. This model was parameterized using an independent database of hydration free energies, making its application to the Minnesota Solvation Database a truly predictive test. For neutral organic solutes in non‐aqueous solvents from the Minnesota Solvation Database (N = 2068 systems), their GAFF2/ABCG2 model achieved an MUE of 0.63 kcal/mol and an RMSE of 0.89 kcal/mol [63]. A key distinction of molecular simulation methods is their ability to explicitly sample and account for conformational changes of the solute in both the gas phase and solution. While molecular simulation can, in principle, provide a more comprehensive physical description by incorporating explicit solvent molecules and dynamic conformational sampling, this comparison highlights that the predictive accuracy of the GAFF2/ABCG2 explicit solvent model, while accounting for dynamic conformational sampling, is comparable to that achieved by the rapid uESE implicit solvation model for the Minnesota Solvation Database, yet at a significantly greater computational expense. This underscores the inherent trade‐off between computational efficiency and comprehensive physical representation, and reinforces the value of uESE for efficient, high‐throughput solvation free energy calculations.

### 3.2 dGsolvDB1 Database

**FIGURE 6:**
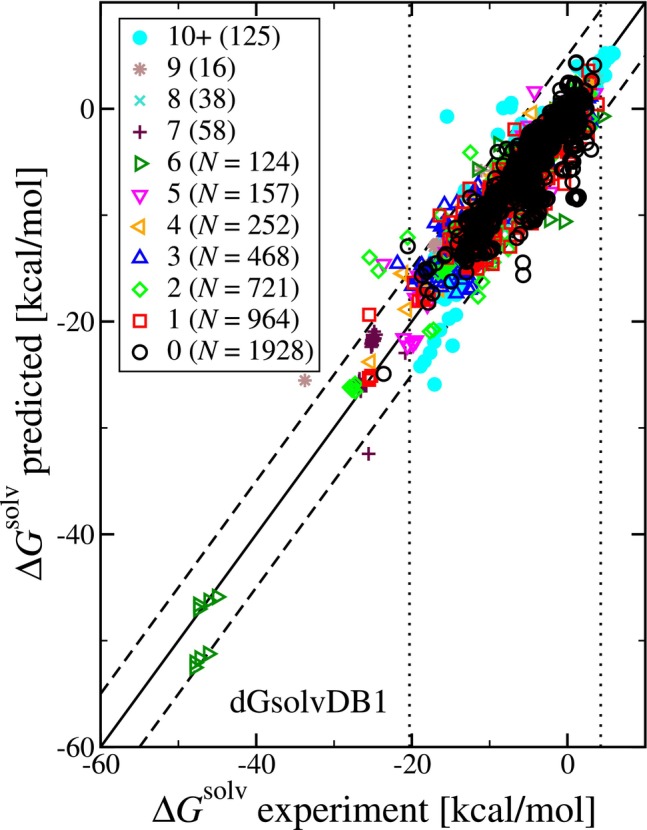
Parity plot of the predicted (uESE set 1) versus experimental solvation free energy ($\Delta G^{\text{solv}}$) from the dGsolvDB1 database. The vertical dotted lines indicate the range of reference values in the Minnesota Solvation Database (−20.3 to 4.28 kcal/mol), and the dashed lines offset from the y=x line illustrate a range of ±5 kcal/mol and are drawn as a reference. The symbol shape and color correspond to the number of torsional angles as indicated in the legend, where the number in parentheses corresponds to the number of systems. The notation 10+ indicates at least 10 torsional angles (specifically, 10 to 26).

We consider next the independent subset of the dGsolvDB1 database [18]. In Figure 6, we first compare the uESE predictions versus experimental solvation free energies ($\Delta G^{\text{solv}}$) from the dGsolvDB1 database. Where the solvent is water (N=1354) and non‐water (N=3497), we have a mean unsigned error (MUE) of 1.30 and 0.89 kcal/mol, respectively, and an overall (N=4851) MUE of 1.00 kcal/mol. The MUE is slightly higher than we obtained for the Minnesota Solvation Database of 0.91 and 0.51 kcal/mol, where the solvent was water (N=388) and non‐water (N=2138), respectively. Interestingly, in both cases, the increase was approximately the same at 0.39 and 0.38 kcal/mol, respectively. However, the number of systems has increased (2526 to 4851) with an increased representation of water as a solvent (15.36% to 27.91%).

The dGsolvDB1 reference data contains 68 systems with values outside the range covered by the Minnesota Solvation Database of −20.3 to 4.28 kcal/mol. For these systems, the overall MUE is 2.69 kcal/mol. For the systems with reference $\Delta G^{\text{solv}}<-20.3$ kcal/mol (N=55), we have an MUE of 3.12 kcal/mol, and for the systems with reference $\Delta G^{\text{solv}}>4.28$ kcal/mol (N=13), we have an MUE of 0.87 kcal/mol. Considering the case where methanol was the solvent (N=161), which was not represented in the Minnesota Solvation Database earlier, we have an MUE of 0.75 kcal/mol. These results suggest the ability to make reasonable predictions with uESE for systems not well represented by the training data.

**FIGURE 7:**
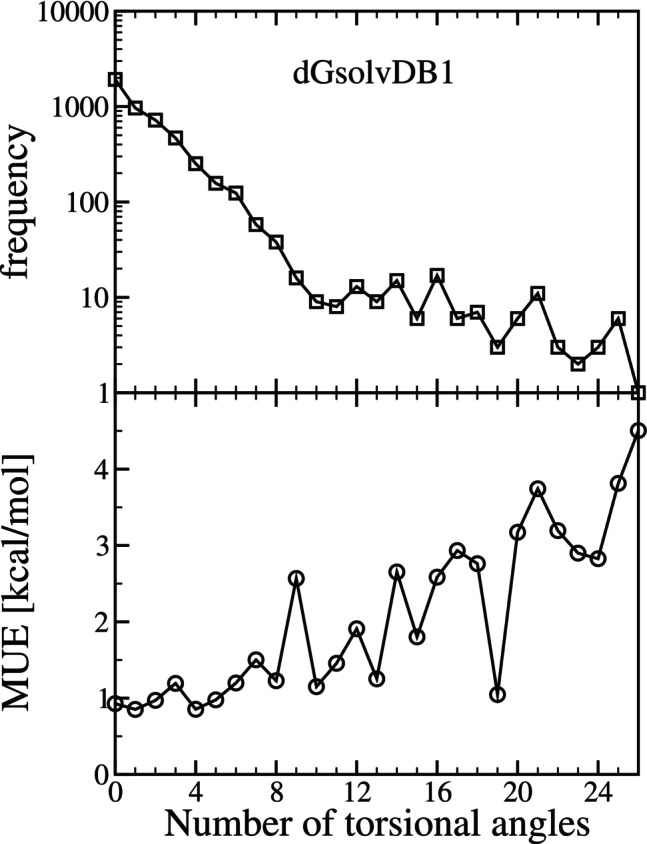
The top pane is the frequency of systems versus the number of torsional angles of the solute, and the bottom pane is the mean unsigned error (MUE) versus the number of torsional angles of the solute. The results correspond predicted (uESE set 1, single conformation) versus experimental solvation free energy ($\Delta G^{\text{solv}}$) from the dGsolvDB1 database.

The effect of the number of torsional angles on the calculation of $\Delta G^{\text{solv}}$ is investigated next in Figure 7. In the top panel, we plot the number (frequency) of solvent–solute pairs versus the number of solute torsional angles. As compared to the Minnesota Solvation Database, while the range of the number of torsional angles increases, the database is still composed primarily of relatively small molecules. Considering the number of systems where the number of solute torsional angles is less than or equal to 6, they make up approximately 98 (2481 of 2526) and 95% (4614 of 4851) of the Minnesota Solvation Database and the dGsolvDB1 database, respectively. With the Minnesota Solvation Database, through the case of 6 torsional angles, the MUE remained below 1 kcal/mol. With the dGsolvDB1 database, through the case of 6 torsional angles, the MUE was below 1 kcal/mol except for the case of 3 torsional angles (N=468, MUE=1.19) and 6 torsional angles (N=124, MUE=1.20). Beyond the case of 6 torsional angles, with the dGsolvDB1 database, the MUE does appear to increase with increasing torsional angle number. However, it is difficult to draw conclusions given the low number of systems. With the Minnesota Solvation Database, the largest MUE was 2.86 kcal/mol, which was for the case of 18 torsional angles (the largest in the database), where N=1. Likewise, with the dGsolvDB1 database, the largest MUE was 4.51 kcal/mol, which was for the case of 26 torsional angles (the largest in the database), where N=1. The dGsolvDB1 system corresponded to didodecyl phthalate (CAS: 2432‐90‐8) in water with a reference value of −5.92 and a uESE prediction of −1.415 kcal/mol. Nonetheless, uESE does capture the correct magnitude and sign, indicating favorable solvation. We do note, however, that for the case of uESE sets 2 (Bolt) and 3 (Bolt), the value of $\Delta G^{\text{solv}}$ does decrease to −6.112 and −7.134 kcal/mol in closer agreement with the reference data for this system. While this does demonstrate the significant effect of structure on the computed $\Delta G^{\text{solv}}$, it is difficult to draw conclusions as it is just a single reference point. Moreover, the only data available for didodecyl phthalate is in water. The conformational effect may be different in different solvents.

Further, for the case of at least one torsional angle, the average value of the set 1 $\Delta G^{\text{solv}}$ relative to the reference (experimental) value is 0.068 kcal/mol (N=2923), comparable to the results with the Minnesota Solvation Database. This is reflected in Figure 6by the accuracy of the uESE predictions that are well distributed above and below the y=x reference line. The overall effect of using multiple conformations would lead to a decrease in the value of $\Delta G^{\text{solv}}$ when the number of torsional angles is greater than or equal to 1. For the case of 0 torsional angles, in Figure 6, we find that the $\Delta G^{\text{solv}}$ values primarily fall within the range covered by the Minnesota Solvation Database. Taking an outlier to be a prediction whose absolute difference from the reference $\Delta G^{\text{solv}}$ is 5 kcal/mol or greater, we have 108 outliers. Of these, 50 correspond to systems with 0 torsional angles. This is interesting as the inclusion of multiple conformations would have no effect in this case.

Within Figure 6, the eight most negative values of $\Delta G^{\text{solv}}$ correspond to 2 isomeric structures of the solute rutin (see Figure S5 in SI‐1) in methanol, ethanol, propanol, and butanol with an average MUE of 2.554 kcal/mol. For the first form of rutin, in all cases, uESE set 1 predicted a $\Delta G^{\text{solv}}$ more negative by an average of 4.55 kcal/mol. For the second form of rutin, in two cases $\Delta G^{\text{solv}}$ is predicted to be more negative by an average of 0.50 kcal/mol, and for the other two cases larger by an average of 0.63 kcal/mol. For this case, the inclusion of multiple conformations results in an overall decrease in $\Delta G^{\text{solv}}$, which would have the effect of increasing MUE.

**FIGURE 8:**
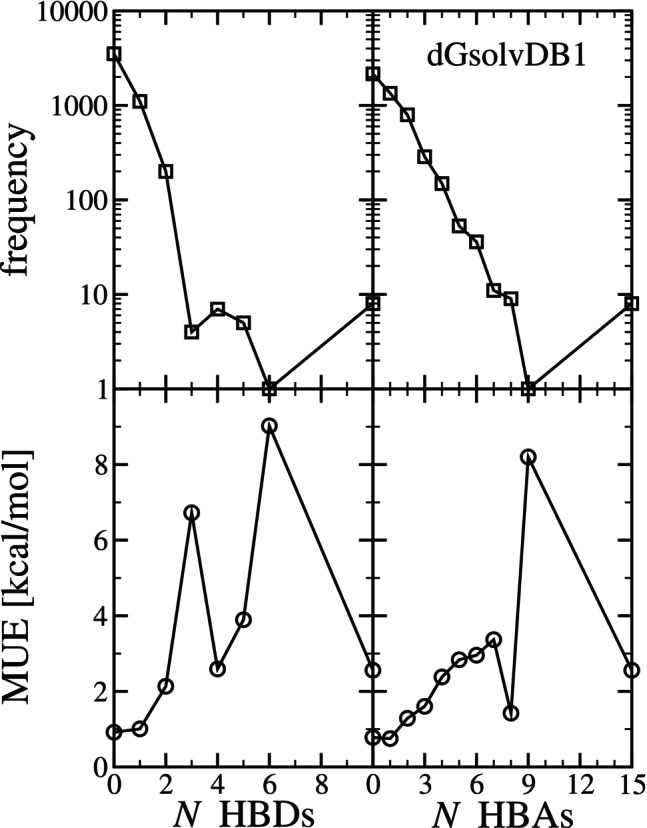
The top row is the frequency of systems versus the number (*N*) of hydrogen bond donor sites (HBDs) and the number (*N*) of hydrogen bond acceptor sites (HBAs) of the solute in columns 1 and 2, respectively. The bottom row is the mean unsigned error (MUE) versus the number (*N*) of hydrogen bond donor sites (HBDs) and the number (*N*) of hydrogen bond acceptor sites (HBAs) of the solute in columns 1 and 2, respectively. The results correspond predicted (uESE set 1, single conformation) versus experimental solvation free energy ($\Delta G^{\text{solv}}$) from the dGsolvDB1 database.

Next, in Figure 8, we investigate the error versus the number of hydrogen bond donors (HBD) and hydrogen bond acceptor (HBA) sites in the solute. Considering first HBD, of the 4851 systems, a majority (3520 systems) have a solute with 0 hydrogen bond donor sites, 1106 systems have a solute with 1 hydrogen bond donor site, 200 systems have a solute with 2 hydrogen bond donor sites, and for all other cases, the count is 10 or less. While the error appears to increase with increasing hydrogen bond donor sites, it is difficult to draw conclusions given the sample composition. The large MUE for the case of 3 and 6 hydrogen bond donor sites corresponds to 4 and 1 systems, respectively. The system with 6 hydrogen bond donating sites corresponds to mannitol (CAS: 69‐65‐8) in water. For this case, the reference $\Delta G^{\text{solv}}$ is −23.62 kcal/mol, and the uESE predictions with sets 1, 2 (Bolt), and 3 (Bolt) correspond to −14.597, −23.299, and −17.993 kcal/mol, respectively. While we again see improvement in this outlier with the inclusion of multiple conformations, it is difficult to draw conclusions, as it is just a single reference point.

Considering next the number of hydrogen bond acceptor sites in the solute, we observe that in general MUE increases with the number of hydrogen bond acceptors, with an outlier for the case of 9 HBA sites. The case of 9 HBA sites corresponds to the single system of the solute depicted in Figure S6 of SI‐1 in water. The reference $\Delta G^{\text{solv}}$ is −33.74 kcal/mol, and the uESE predictions with sets 1, 2 (Bolt), and 3 (Bolt) correspond to −25.539, −27.952, and −25.144 kcal/mol, respectively. We find that for this case, the effect of including multiple conformations is less significant. However, it is difficult to draw firm conclusions from the results. The majority of the systems have 0 (N=2158) or 1 (N=1345) HBA site. Similar to the case of the Minnesota Solvation Database, the range of the number of hydrogen bond acceptor sites is greater than the range of the number of hydrogen bond donor sites. Nonetheless, we again find that the error is comparable.

Within SI‐1, a discussion is also provided on the error vs the number of non‐hydrogen (intramolecular) bonds (Figure S3) and atoms (Figure S4) as a measure of the size of the solute, with the results consistent with those presented already. We also provide detailed results in spreadsheets in SI‐2 accompanying the electronic version of this manuscript. We tabulate all of the predictions and descriptors used to characterize the solute molecules, and a summary of the error broken down by solvent (including non‐water) and uESE solvent class. We additionally provide a summary of the error based on the solute descriptor (torsional angles, atoms and bonds, hydrogen bond donor sites, and hydrogen bond acceptor sites). All of the errors based on the solute descriptor are provided overall, when water is the solvent, and for non‐water solvents.

#### 3.2.1 Multiple Conformations

While our analysis of the dGsolvDB1 database focused on set 1 (single conformation), a few cases were noted wherein the use of multiple conformations offered improved predictions of $\Delta G^{\text{solv}}$. Sets 2, 3, and 23 predictions were additionally made for the dGsolvDB1 database. However, there were two extreme outliers with nonphysical predictions, which has caused a pause in the use of multiple conformations. The chemical structure of the two solute molecules is provided in Figure S7 of SI‐1. For 3‐chloropyridine (CAS: 626‐60‐8) in water, sets 2 and 3 predict a $\Delta G^{\text{solv}}$ of 413.15 kcal/mol, compared to a reference value of −4.01 kcal/mol. On the other hand, the set 1 prediction was −4.138 kcal/mol. The result is puzzling as Open Babel generates only a single conformation for 3‐chloropyridine, with the starting conformation taken from set 1. The structure generation and calculations were repeated, but the results did not change. Within uESE, the total solvation free energy, $\Delta G^{\text{solv}}$, is written as a sum of an electrostatic (COSMO) contribution resulting from solute–solvent intermolecular electrostatic interactions and a second “correction” term that accounts for cavity formation and dispersion and repulsion interactions [64, 65, 66, 67, 68, 69, 70, 71, 72, 73, 74, 75, 76, 77, 78, 79, 80, 81, 82, 83, 84, 85, 86, 87, 88, 89, 90, 91, 92, 93, 94, 95]. Comparing sets 1 and 2, the electrostatic contribution to the total $\Delta G^{\text{solv}}$ is −4.131 and −3.151 kcal/mol, respectively, while the “correction” term is −0.007 and 416.301 kcal/mol, respectively. For set 1, the solvent‐independent total cavity (van der Waals) area and volume computed during the uESE calculations are 121.250 Å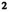 and 93.438 Å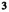, respectively, which agree well with the set 2 values of 121.439 Å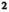 and 93.541 Å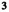, respectively. Furthermore, we find that the sets 1 and 2 CM5 partial atomic charges are virtually unchanged, with the charge of 5 (of 11) sites changing by one ten‐thousandth. The results again emphasize that subtle changes in geometry can have a large impact on the resulting $\Delta G^{\text{solv}}$. This is the same as was observed for the two outliers in comparing sets 0 and 1 with the Minnesota Solvation Database.

The second outlier is butylbenzene (CAS: 104‐51‐8), which is an outlier for set 2 but not set 1 or 3. For this case, Open Babel generated 10 unique conformations. The prediction for $\Delta G^{\text{solv}}$ in water is −0.848 kcal/mol and is in good agreement with the reference value of −0.37 kcal/mol. However, for all other cases (butylbenzene in methanol, ethanol, butanone, acetonitrile, heptane, hexadecane, and tetrahydrofuran) $\Delta G^{\text{solv}}$ is greatly underpredicted. For the case of butylbenzene in methanol, the predicted $\Delta G^{\text{solv}}$ is −2202 kcal/mol as compared to the reference value of −5.81 kcal/mol.

#### 3.2.2 Additional Benchmarking

The filtered dGsolvDB1 database serves as an independent validation set for uESE, as its experimental solvation free energies are entirely distinct from the Minnesota Solvation Database used in uESE's parameterization. dGsolvDB1 aggregates values from diverse sources, including the CompSol [98] and FreeSolv [18] databases, alongside numerous publications from Abraham, Acree, and co‐workers [99, 100]. To provide context for uESE's predictive performance, we focus on the widely used FreeSolv database, a subset of dGsolvDB1 comprising 642 hydration free energies. For these 642 FreeSolv systems, uESE with set 1 (single MMFF94 conformation) yields a mean unsigned error (MUE) of 0.97 kcal/mol. We note that for the 311 FreeSolv systems also found in other dGsolvDB1 sources, the dGsolvDB1 authors reported the average, which we used throughout this work.

We first compare uESE's performance to explicit solvent molecular simulation approaches, which aim for a more comprehensive physical representation but at a greater computational cost. Wang and co‐workers, in their recent work on the ABCG2 charge model for GAFF2 [101], reported predictive accuracies for all 642 FreeSolv systems. Using GAFF2 with AM1‐BCC charges, they obtained an MUE of 1.22 kcal/mol. With RESP charges (which, like CM5 charges, require an electronic structure single‐point energy calculation), the MUE was 1.06 kcal/mol. Their novel ABCG2 charge model yielded an MUE of 0.57 kcal/mol for the full 642 FreeSolv systems. However, it is important to note that the ABCG2 model was parameterized on a subset of 441 FreeSolv systems, achieving an MUE of 0.37 kcal/mol on this training set, while the true predictive performance on the remaining 201 independent systems resulted in an MUE of 1.02 kcal/mol. This highlights the distinction between reproduction within a training set and true external prediction for their model.

Further benchmarks from explicit solvent molecular simulations on the FreeSolv database include the foundational work by Mobley and co‐workers, who, during the development of the database, reported an MUE of 1.12 kcal/mol for all 642 systems using the GAFF force field with AM1‐BCC charges [102]. Similarly, Boresch and co‐workers, employing explicit solvent molecular simulations with CGENFF and OpenFF 2.0 force fields, achieved MUEs of 1.12 kcal/mol (for 621 systems) and 1.01 kcal/mol (for 589 systems), respectively [103, 104]. All of these explicit solvent results are purely predictive on the FreeSolv database. Notably, Boresch and co‐workers observed that force fields like GAFF, CGENFF, and OpenFF tend to over‐predict solvation free energies on average, indicating a solute that is modeled as too hydrophobic. In the work of Reference [103], GAFF was used with two atoms‐in‐molecules approaches to obtain partial atomic charges. To account for polarization effects, the charges were derived from the solute's electron density computed with an implicit solvent model, and the energy required to polarize the solute was added to the free energy cycle. Despite this advanced treatment and increased computational cost, the predictions were found to be inferior to the use of AM1‐BCC charges.

In the realm of efficient, high‐throughput predictions using alternative implicit‐like methods, molecular density functional theory (MDFT) in the hypernetted chain approximation coupled with revised pressure correction has also been applied to hydration free energies. Reference [104] reported an MUE of 1.07 kcal/mol for 619 FreeSolv systems, with 23 systems excluded due to non‐convergence. This method, which also employed a single solute structure, was noted to have an average calculation time of 1 min 53 s per system on a single CPU thread.

Collectively, these comparisons demonstrate that uESE provides comparable, and in many cases, superior predictive accuracy for solvation free energies on an independent validation set (FreeSolv), especially when contrasted with explicit solvent molecular simulations, while maintaining a significantly lower computational cost. This further underscores uESE's utility for efficient, high‐throughput computational screening.

While our primary benchmarking focused on the widely used Minnesota Solvation and FreeSolv databases, it is important to recognize the recent work by Fain and co‐workers [105] that has demonstrated the ability to further improve the accuracy achieved using molecular simulation. In Reference [97], the authors developed the polarizable ARROW force field, which was fit entirely to ab initio calculations. For a set of 85 hydration free energies of neutral organic compounds, they achieved an MUE of 0.78 kcal/mol using molecular dynamics (MD). However, with the use of the Path Integral formulation of nuclear motion (PIMD) to include nuclear quantum effects, the MUE decreased to an exceptional 0.20 kcal/mol. The authors also considered a set of 40 solvation free energies of neutral organic compounds in cyclohexane, where the MUE decreased from 0.45 kcal/mol to 0.30 kcal/mol in going from MD to PIMD. This exceptional accuracy came with a significant increase in computational cost, approximately a factor of 8. In a subsequent work [98], the authors constructed a neural network term to augment the intermolecular interactions, an approach expected to lead to even greater performance.

To provide context, with our computationally efficient uESE model, we obtain an MUE of 1.297 kcal/mol for water (N=1354) and 0.738 kcal/mol for cyclohexane (N=42) on the dGsolvDB1 database. This comparison reinforces the inherent trade‐off between computational efficiency and comprehensive physical representation. While these advanced explicit solvent methods are pushing the boundaries of accuracy, uESE remains a valuable tool for efficient, high‐throughput solvation free energy predictions where this level of precision is not required. It is a paradigm we expect may change in the future with continued improvements in computational resources.

## 4 Summary and Conclusion

In the present study, we sought to assess the feasibility of performing high‐throughput solvation free energy calculations using electronic structure calculations, here using the uESE continuum solvation model, by leveraging computationally efficient solute structure generation via molecular mechanics. The ultimate goal would be to incorporate solvation free energies as descriptors in physics‐informed (or chemistry/theoretically‐informed) machine learning models. We hypothesized that the rapid generation of solute geometries using MMFF94, coupled with uESE's use of CM5 partial charges (known for their reduced conformational dependence), could offer a viable route for large‐scale solvation free energy predictions, potentially circumventing the need for computationally intensive high‐level geometry optimizations.

Our investigation on the Minnesota Solvation Database revealed that using a single low‐energy conformation generated with the MMFF94 force field (set 1) yielded predictive accuracy comparable to, and in some cases slightly better than, using rigid gas‐phase optimized geometries provided within the database (set 0). This key finding suggests that for many systems, the computationally inexpensive MMFF94 method can provide sufficiently accurate solute structures for uESE calculations.

Our comprehensive assessment revealed a notable and practical finding: The explicit use of multiple solute conformations, generated through molecular mechanics (sets 2 and 3), did not consistently improve the predictive accuracy of the uESE model for solvation free energies when compared to the single MMFF94 conformation approach on the Minnesota Solvation Database. This suggests that for continuum solvation models like uESE, particularly those employing CM5 charges with their inherently weaker conformational dependence, computationally more intensive conformational sampling may not be essential. We hypothesize that this may also be due to the uESE model being parameterized on single, low‐energy structures, making it optimally suited for such input. While we identified cases in the dGsolvDB1 database that showed improved predictions with multiple conformations, it is difficult to draw a general conclusion as dGsolvDB1 consisted primarily of relatively small molecules. Likewise, cases were identified where the predictions were worse. In general, the effect of considering multiple conformations is to decrease the value of the predicted solvation free energy. Given these findings, we suggest that using a single, optimized conformation for the solute is a robust and highly efficient approach for uESE predictions, which is better aligned with the goal of high‐throughput calculations and makes this model even more attractive for large‐scale applications.

Evaluation of uESE (using single MMFF94 conformations) on the independent dGsolvDB1 database demonstrated reasonable predictive ability, with a slight increase in the overall mean unsigned error compared to the Minnesota Solvation Database. Notably, uESE showed some capacity to predict solvation free energies for systems with experimental values outside the training data range and for neutral molecules in methanol, which were not present in our filtered Minnesota Solvation database, indicating a degree of generalizability. Analysis of error trends with molecular descriptors (number of torsional angles, hydrogen bond donors and acceptors, and size) revealed some correlations, but these were often influenced by the distribution of molecules within the datasets.

Beyond our internal assessment, this study contextualized uESE's performance against other established methods for solvation free energy prediction. While models like SM12 and openCOSMO‐RS showed slightly lower MUEs on the Minnesota Solvation Database, it is important to recall that all these implicit models were parameterized using this same dataset, making direct conclusions on their true predictive superiority challenging. More importantly, when evaluated on the independent FreeSolv subset of dGsolvDB1, uESE demonstrated predictive accuracy that was comparable to, and in several cases, superior to results obtained from far more computationally intensive explicit solvent molecular simulation approaches, including those using sophisticated charge models like ABCG2 or advanced polarization treatments. These explicit solvent methods, while offering detailed physical representation and conformational sampling, typically incur a significantly higher computational expense. Lastly, comparison to other high‐throughput approaches like molecular density functional theory underscored uESE's favorable balance of accuracy and computational efficiency.

In conclusion, our findings suggest that a workflow employing a single solute structure generated via molecular mechanics (MMFF94) coupled with the uESE continuum solvation model presents a promising approach for efficient, high‐throughput solvation free energy calculations. The comparable performance of MMFF94‐generated single conformations to more computationally demanding geometry generation using electronic structure calculations supports the potential of this strategy. While considering multiple conformations did not offer a general improvement in accuracy in this study, further investigation into tailored conformational sampling strategies for specific classes of flexible molecules may still be beneficial. Future work could also explore the performance of this approach with other force fields and on even larger and more diverse datasets.

## Author Contributions

**Andrew S. Paluch:** conceptualization, data curation, formal analysis, funding acquisition, investigation, methodology, project administration, resources, software, validation, visualization, writing – original draft, writing – review and editing. **Jeffrey G. Ethier:** conceptualization, funding acquisition, project administration, visualization, writing – original draft, writing – review and editing. **Vikas Varshney:**conceptualization, funding acquisition, project administration, visualization, writing – original draft, writing – review and editing.

## Conflicts of Interest

The authors declare no conflicts of interest.

## Supporting information




**Data S1**: (SI‐1) contains a discussion of the error versus the number of non‐hydrogen (intramolecular) bonds and atoms as a measure of the size of the solute, images of chemical structures of interest, and a note on the use of the average absolute percent deviation (AAPD).

**Data S1**: (SI‐2) contains spreadsheets wherein we tabulate all of the predictions and descriptors used to characterize the solute molecules, and a summary of the error broken down by solvent (including non‐water) and uESE solvent class. We additionally provide a summary of the error based on the solute descriptor (torsional angles, atoms and bonds, hydrogen bond donor sites, and hydrogen bond acceptor sites). All of the errors based on the solute descriptor are provided overall, when water is the solvent, and for non‐water solvents.

## Data Availability

The data that support the findings of this study are available in the Supporting Information of this article.

## Endnotes

^1^
Considering the case of octanol‐water, the mutual solubility of water in 1‐octanol is 0.207 mole fracs, while the mutual solubility of 1‐octanol in water is $0.703\times 10^{-4}$ mole fracs [59] It is therefore reasonable to take the water‐rich phase to be pure water. However, the water in the octanol‐rich phase is not negligible. Chen and Siepmann [60] have used Monte Carlo simulations with advanced sampling techniques to show that pure 1‐octanol contains microscale heterogeneities consisting of polar (hydroxyl head group) and non‐polar (alkyl tail) domains. In water‐saturated 1‐octanol, they found that considerably more large hydrogen bond aggregates (polar domains) exist. This change in structure in turn may have a significant effect on solvation free energy [61, 62]. This motivated the exclusion of solvation free energies derived from partitioning data.

^2^
To be clear, the dGsolvDB database was filtered to contain only the 92 solvents for which uESE is parameterized. Data was available for all but 13 solvents: o‐dichlorobenzene, o‐nitrotoluene, sulfolane, bromooctane, chlorohexane, decalin, dimethylpyridine, 1‐fluorooctane, hexadecyl‐iodide, diphenyl ether, t‐butylbenzene, tributyl‐phosphate and xylene. We then removed systems containing Ar, , Ge, diatomic hydrogen, He, Hg, Kr, Ne, Rn, Xe, Sn. These systems were removed as they are unable to be modeled using MMFF94.

^3^
To be clear, as an example, the number of torsional angles not involving hydrogen atoms for butane is 1 (C‐C‐C‐C). Additionally, aromatic rings are taken to be rigid, planar structures. Therefore, the number of torsional angles in benzene is 0.

^4^
Note that the terms mean unsigned error (MUE) and mean absolute error (MAE) are often used interchangeably and are equivalent.
