# Supplementary material for: Leveraging Molecular Mechanics With the uESE Continuum Solvation Model for Efficient Solvation Free Energy Prediction: Impact of Conformation and Extensive Validation
Source: J Comput Chem. 2025 Oct 22;46(28):e70252. doi: 10.1002/jcc.70252 (PMC12543295; doi:10.1002/jcc.70252)
Supplement: Supplementary file 1 — Data S1: (SI‐1) contains a discussion of the error versus the number of non‐hydrogen (intramolecular) bonds and atoms as a measure of the size of the solute, images of chemical structures of interest, and a note on the use of the average absolute percent deviation (AAPD). [file JCC-46-0-s002.pdf]

# Supporting Information 1 for "Leveraging Molecular Mechanics with the uESE Continuum Solvation Model for Efficient Solvation Free Energy Prediction: Impact of Conformation and Extensive Validation"

Andrew S. Paluch[a], Jeffrey G. Ethier[b], Vikas Varshney[b]

[a]*Department of Chemical, Paper and Biomedical Engineering, Miami University, Oxford, Ohio 45056, USA*
[b]*Materials and Manufacturing Directorate, Air Force Research Laboratory, Wright-Patterson Air Force Base, Ohio 45433 USA*

---

**List of Figures**

S1 Error versus the number of non-hydrogen (intramolecular) bonds for the Minnesota Solvation Database. . . . . . . . . . 4
S2 Error versus the number of non-hydrogen atoms for the Minnesota Solvation Database. . . . . . . . . . . . . . . . . . . . . . 5
S3 Error versus the number of non-hydrogen (intramolecular) bonds for the dGsolvDB1 database. . . . . . . . . . . . . . . 8
S4 Error versus the number of non-hydrogen atoms for the dGsolvDB1 database. . . . . . . . . . . . . . . . . . . . . . . . 9
S5 Chemical structure of rutin. . . . . . . . . . . . . . . . . . . . 10
S6 Chemical structure of the outlier with 9 hydrogen bond acceptor (HBA) sites. . . . . . . . . . . . . . . . . . . . . . . . 11
S7 Chemical structure of 3-chloropyridine and butylbenzene. . . 12

*Email address:* PaluchAS@MiamiOH.edu (Andrew S. Paluch)

## Results and Discussion

*Note on Average Absolute Percent Deviation*

In the figures within the main manuscript, we have not included the average absolute percent deviation (AAPD). The challenge is that there are a small number of systems with large values of AAPD that skew the results in the dGsolvDB1 database. Within the dGsolvDB1 database there are a total of 38 systems with reference solvation free energies between −0.1 and 0.1 kcal/mol, of which 5 have a value of 0 kcal/mol. Excluding the reference systems of 0 kcal/mol, the largest AAPD is for beta-selinene (CAS: 17066-67-0) in water with an AAPD of 6520%. The reference solvation free energy of beta-selinene is −0.05 kcal/mol with a uESE prediction of −3.31 kcal/mol, corresponding to a difference of 3.26 kcal/mol. Detailed results are tabulated in spreadsheets in Supporting Information 2 (SI-2) accompanying the electronic version of the manuscript.

*Minnesota Solvation Database*

As a final comparison, in fig. S1 and fig. S2 we plot the error versus the number of non-hydrogen (intramolecular) bonds and atoms, respectively, as a measure of the size of the solute. The large AAPD at low bond/atom numbers is caused by the error in a few systems with small experimental values of $\Delta G^{\mathrm{solv}}$. For example, the largest observed absolute percent error is for the system ethyne in water, where ethyne has two non-hydrogen atoms and one non-hydrogen bond. The reference $\Delta G^{\mathrm{solv}}$ is –0.01 kcal/mol while the set 1 prediction is –1.187 kcal/mol, resulting in a percent error of 11770%. Both the MUE and AAPD are relatively larger at large bond/atom numbers, however, we are again limited in that there are few systems. The

Minnesota Solvation Database is limited in that it is mostly composed of relatively small solute molecules.

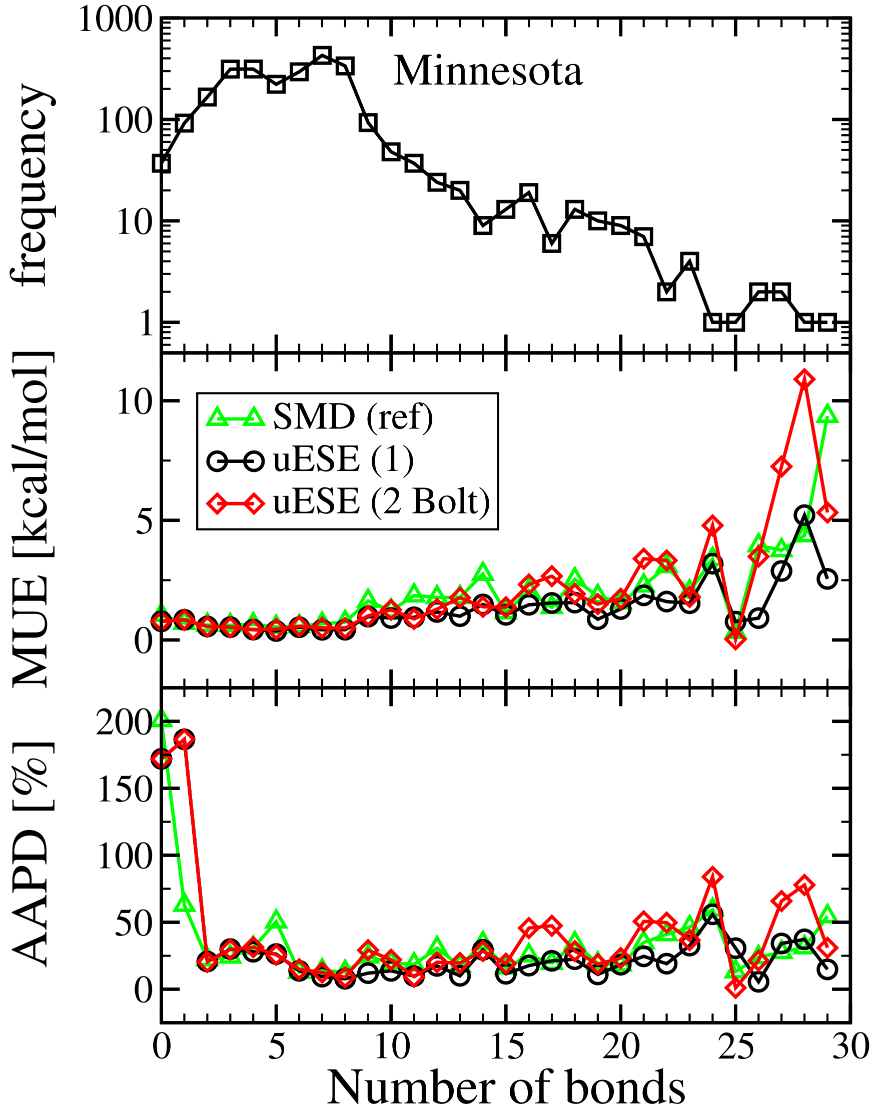


Figure S1: The top pane is the frequency of systems versus the number of (intramolecular) bonds not involving hydrogen in the solute. In the bottom two panes are plotted the mean unsigned error (MUE) or average absolute percent deviation (AAPD) versus the number of (intramolecular) bonds not involving hydrogen in the solute. Results are presented for the SMD reference ("ref") calculations [1, 2], uESE set "1" (single conformation), and uESE set "2 Bolt" (multiple conformations, RMSD diversity, Boltzmann weighted) versus reference values from the Minnesota Solvation Free Energy Database.

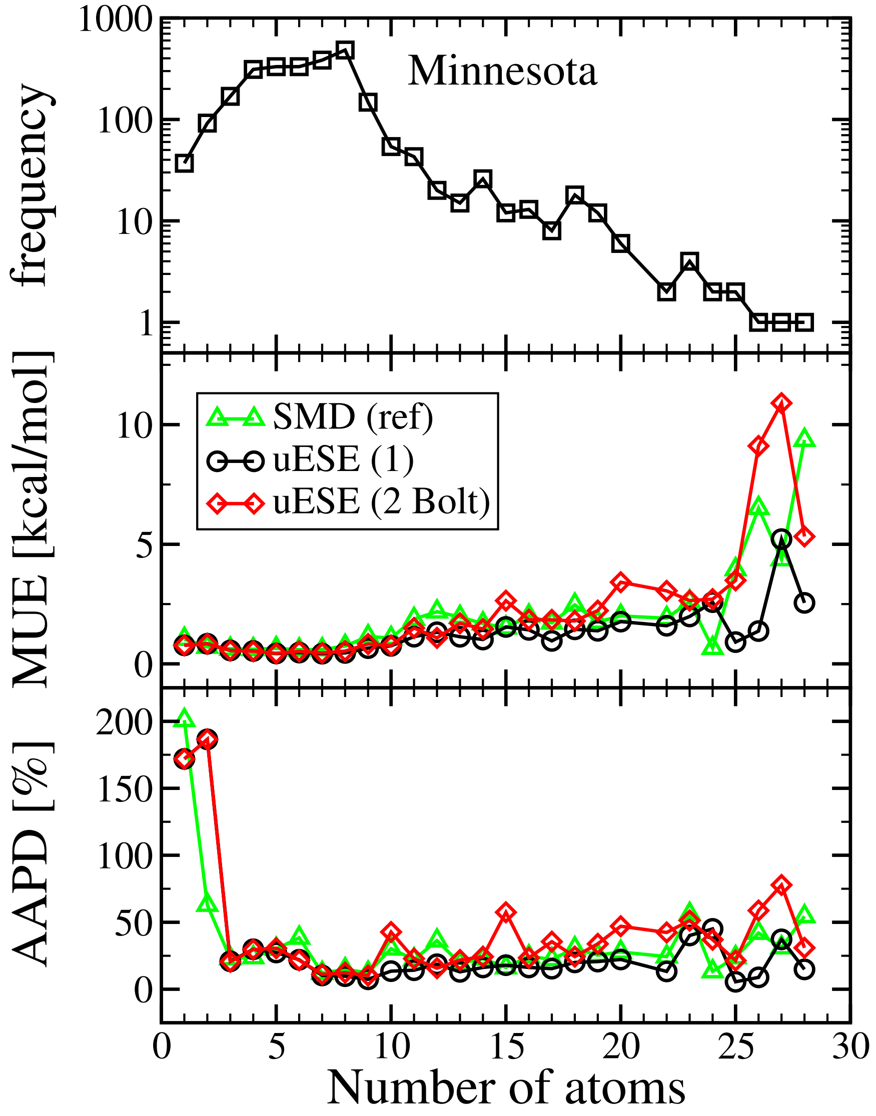


Figure S2: The top pane is the frequency of systems versus the number of non-hydrogen atoms in the solute. In the bottom two panes are plotted the mean unsigned error (MUE) or average absolute percent deviation (AAPD) versus the number of non-hydrogen atoms in the solute. Results are presented for the SMD reference ("ref") calculations [1, 2], uESE set "1" (single conformation), and uESE set "2 Bolt" (multiple conformations, RMSD diversity, Boltzmann weighted) versus reference values from the Minnesota Solvation Free Energy Database.

*dGsolvDB1*

As a final comparison, in fig. S3 and fig. S4 we investigate the error versus the number of non-hydrogen (intramolecular) bonds and atoms, respectively, as a measure of the size of the solute. Considering first the number of bonds, through the case of 20 bonds the MUE remains below 2 kcal/mol; this performance is similar to the results with the Minnesota Solvation Database. Beyond 20 bonds, the MUE fluctuates, but we note that we only have 191 total systems with more than 20 bonds. The largest case corresponds to 47 bonds, for which we have 2 isomeric structures of the solute rutin (see fig. S5) in methanol, ethanol, propanol, and butanol with an average MUE of 2.554 kcal/mol. For the first form of rutin, in all cases uESE set 1 predicted a $\Delta G^{\text{solv}}$ more negative by an average of 4.55 kcal/mol. For the second form of rutin, in two cases $\Delta G^{\text{solv}}$ is predicted more negative by an average of 0.50 kcal/mol, and for the other two cases larger by an average of 0.63 kcal/mol. For this case the inclusion of multiple conformations results in an overall decrease in $\Delta G^{\text{solv}}$, which would have the effect of increasing MUE. The case of rutin also corresponds to eight most negative values of $\Delta G^{\text{solv}}$ within the dGsolvDB1 database, as discussed in the main manuscript. Interestingly, while rutin has the most bonds, it only has 6 torsional angles. As a reminder, we exclude torsional angles involving hydrogen atoms and aromatic rings are assumed planar (or rigid).

For the case of the number of atoms, we first note that we have a peak for the case of two atoms, which is also reflected in the peak for the case of one bond. These systems are comprised primarily of gases, including many diatomic gases. Overall, we again find that overall MUE increases with an increasing number of atoms. Through the case of 19 non-hydrogen (in-

tramolecular) atoms, the average MUE remains below 2 kcal/mol. Beyond 19 atoms the average MUE fluctuates, but we again note that we have a relatively small number of systems ($N = 193$) reflected in this case.

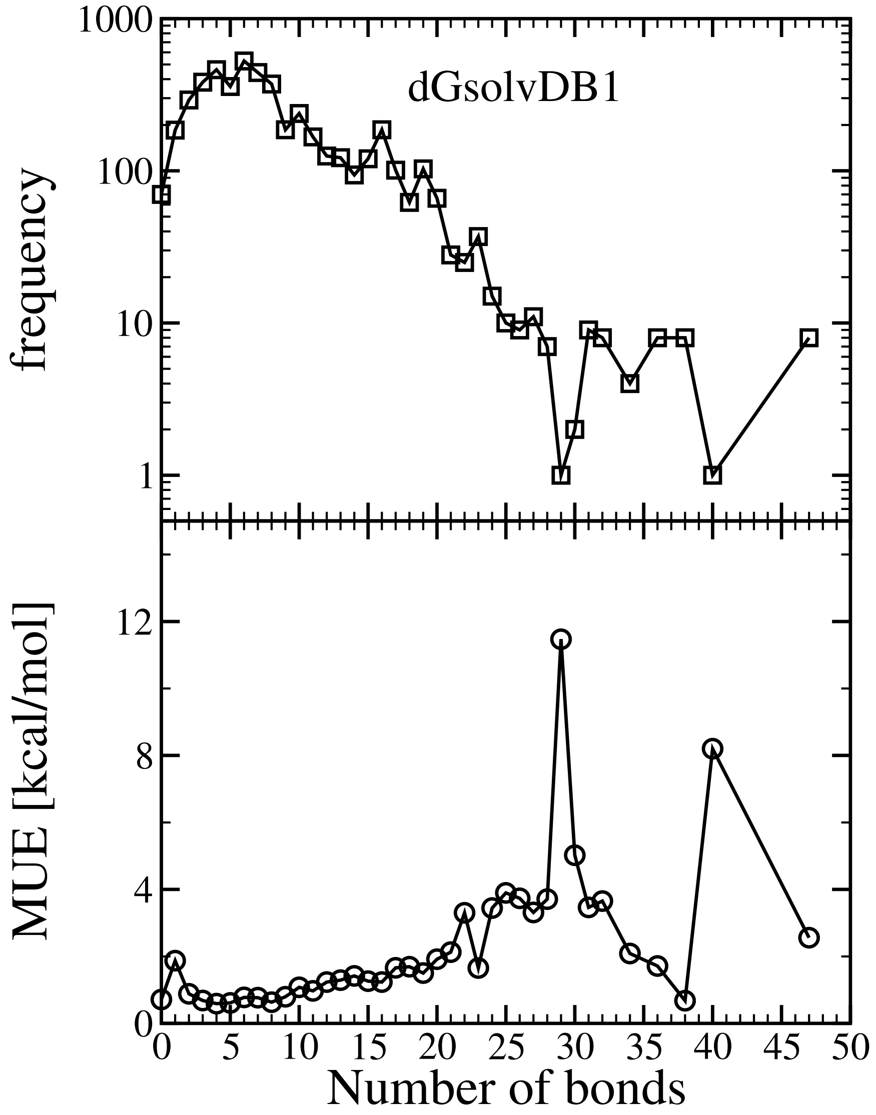


Figure S3: The top pane is the frequency of systems versus the number of (intramolecular) bonds not involving hydrogen of the solute, and the bottom pane is the mean unsigned error (MUE) versus the number of (intramolecular) bonds not involving hydrogen of the solute. The results correspond predicted (uESE set "1", single conformation) versus experimental solvation free energy ($\Delta G^{\text{solv}}$) from the dGsolvDB1 database.

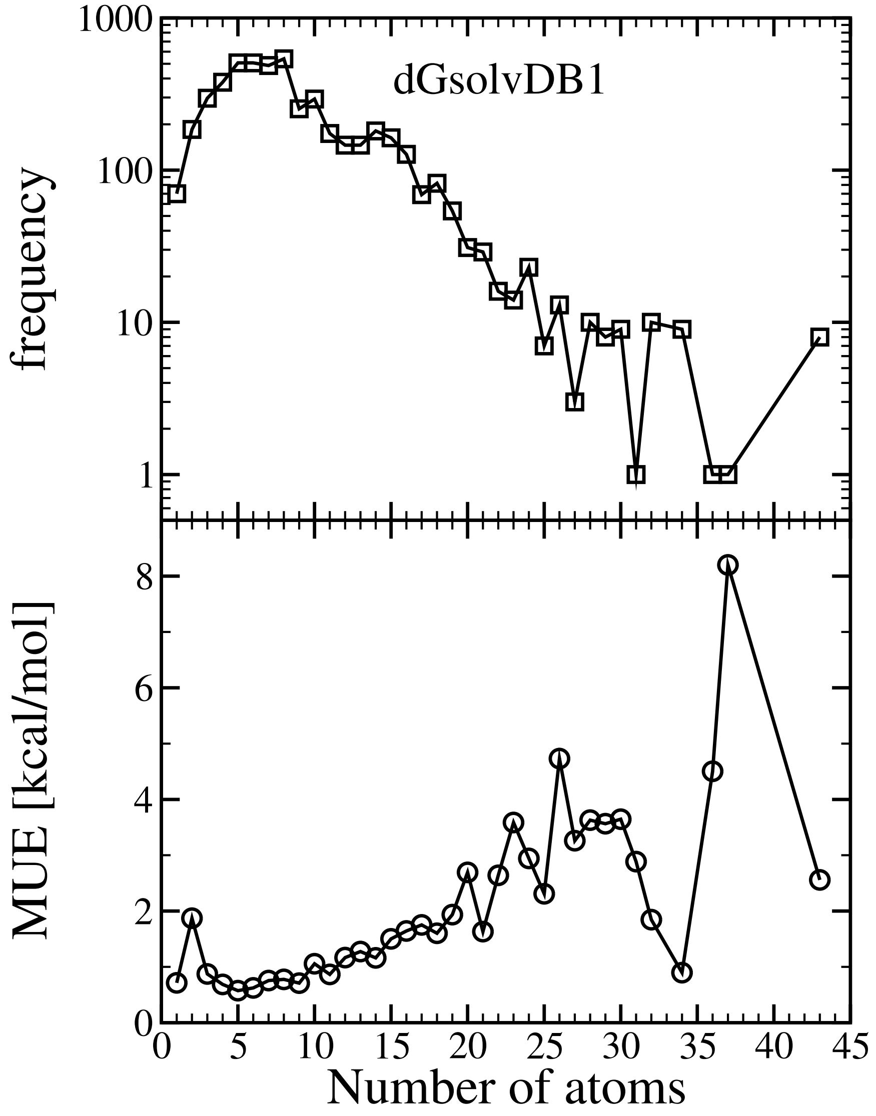


Figure S4: The top pane is the frequency of systems versus the number of non-hydrogen atoms of the solute, and the bottom pane is the mean unsigned error (MUE) versus the number of non-hydrogen atoms of the solute. The results correspond predicted (uESE set "1", single conformation) versus experimental solvation free energy ($\Delta G^{\mathrm{solv}}$) from the dGsolvDB1 database.

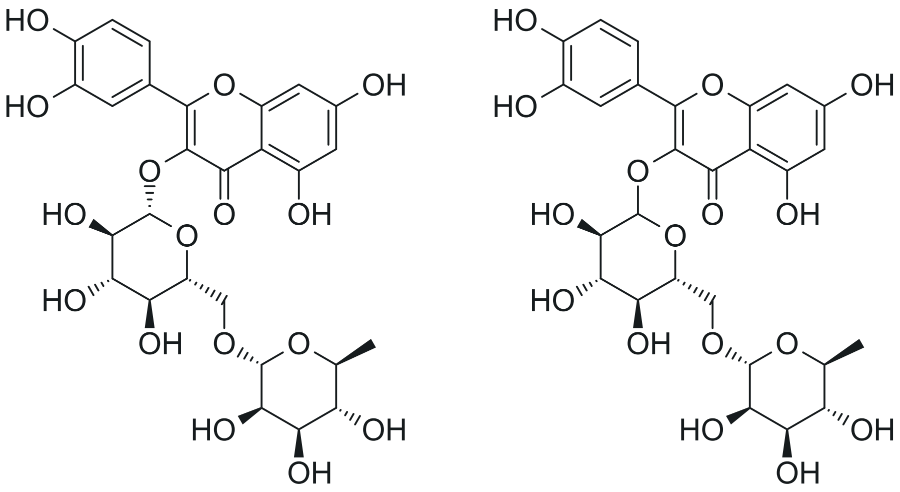

rutin

Figure S5: Chemical structure of rutin (CAS: 153-18-4) corresponding to the solute with the largest number of bonds (47).

*Additional Structures of Interest*

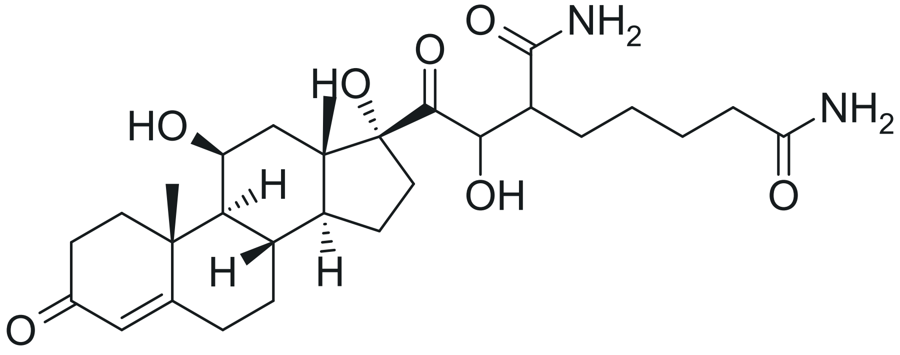


Figure S6: Chemical structure of the outlier with 9 hydrogen bond acceptor (HBA) sites.

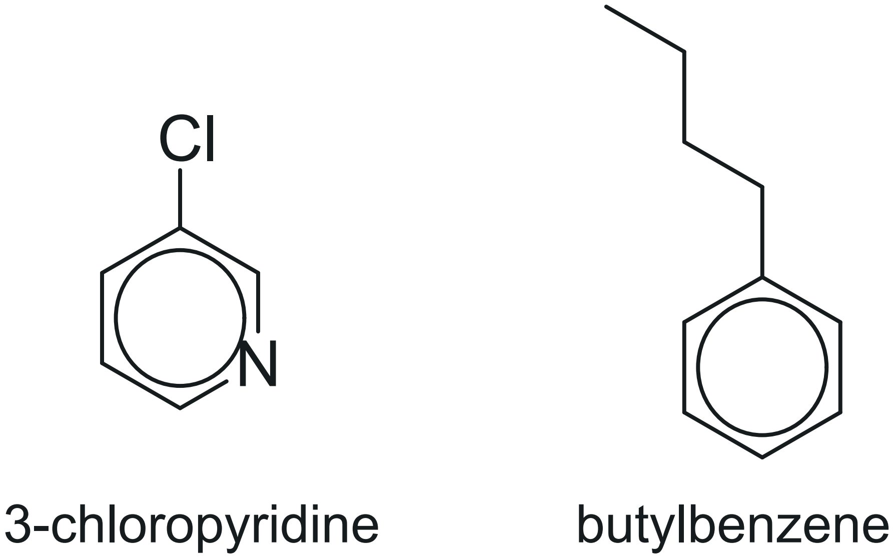


Figure S7: Chemical structure of 3-chloropyridine (CAS: 626-60-8) and butylbenzene (CAS: 104-51-8), outliers when using multiple conformations.

## References

[1] S. F. Vyboishchikov, A. A. Voityuk, Fast non-iterative calculation of solvation energies for water and non-aqueous solvents, J. Comput. Chem. 42 (2021) 1184–1194.

[2] A. A. Voityuk, S. F. Vyboishchikov, Fast and accurate calculation of hydration energies of molecules and ions, Phys. Chem. Chem. Phys. 22 (2020) 14591–14598.
