# Supplementary material for: Leveraging Molecular Mechanics With the uESE Continuum Solvation Model for Efficient Solvation Free Energy Prediction: Impact of Conformation and Extensive Validation
Source: J Comput Chem. 2025 Oct 22;46(28):e70252. doi: 10.1002/jcc.70252 (PMC12543295; doi:10.1002/jcc.70252)
Supplement: Supplementary file 2 — Data S1: (SI‐2) contains spreadsheets wherein we tabulate all of the predictions and descriptors used to characterize the solute molecules, and a summary of the error broken down by solvent (including non‐water) and uESE solvent class. We additionally provide a summary of the error based on the solute descriptor (torsional angles, atoms and bonds, hydrogen bond donor sites, and hydrogen bond acceptor sites). All of the errors based on the solute descriptor are provided overall, when water is the solvent, and for non‐water solvents. [file JCC-46-0-s001.zip › description_of_data.docx]

Supporting Information 2 for “Leveraging Molecular Mechanics with the uESE Continuum Solvation Model for Efficient Solvation Free Energy Prediction: Impact of Conformation and Extensive Validation”

# Andrew S. Paluch*^,a^, Jeffrey G. Ethier^b^ and Vikas Varshney^b^

## [a] Department of Chemical, Paper and Biomedical Engineering, Miami University, Oxford, Ohio 45056, USA [b] Materials and Manufacturing Directorate, Air Force Research Laboratory, Wright-Patterson Air Force Base, Ohio 45433 USA [*] [PaluchAS@MiamiOH.edu](mailto:PaluchAS@MiamiOH.edu)

Supporting Information 2 contains Excel spreadsheets wherein we tabulate all of the predictions and descriptors used to characterize the solute molecules, and a summary of the error broken down by solvent (including non-water) and uESE solvent class. We additionally provide a summary of the error based on the solute descriptor (torsional angles, atoms and bonds, hydrogen bond donor sites, and hydrogen bond acceptor sites). All of the errors based on the solute descriptor are provided overall, when water is the solvent, and non-water solvents. Note, we have not included the values of the reference solvation free energies from the Minnesota Solvation Database version 2020 due to the license under which the values are published.

For the summary of errors for each descriptor, in addition to the descriptor count (e.g., the number of atoms), we provide the: frequency or number of systems (***N***), mean unsigned error (or mean absolute error, **MUE**), root mean squared error (**RMSE**), average absolute percent deviation (**AAPD**), and ***R***^2^ value.

The descriptor counts were obtained directly from Open Babel 3.0.0 using the flag --append “atoms bonds rotors HBD HBA1 HBA2” to append the frequency of occurrence of each descriptor to the provided molecular structure. (MW could also be added to the list to determine the molecular weight.) In doing this we note:

1. **atoms**: The atom count does NOT include hydrogen atoms.
2. **bonds**: The bond count does NOT include any bonds involving hydrogen.
3. **rotors**: The term rotor and torsional angle are used interchangeably. The count does NOT include any torsional angles involving hydrogen. As an example, butane has a single torsional angle (C-C-C-C). Additionally, aromatic rings are assumed planar (or rigid). For this case, benzene has 0 torsional angles.
4. **HBA1 and HBA2**: Open Babel has two descriptors corresponding to the number of hydrogen bond acceptor sites. The findings of this work was consistent regardless of which descriptor was used.

## Minnesota

The folder contains all of the results for the Minnesota Solvation Database in the 21 spreadsheets below.

1. **minnesota_all_data_solvation_fe** - The tab “MNSol_uESE” contains the results from the present study, and “MNSol_uESE+SMD-ref” contains the reference uESE and SMD predictions.
2. **minnesota_error_atom** - Summary of error for atom descriptor, with each tab corresponding to a different set of predictions.
3. **minnesota_error_atom_nonwater** - Summary of error for atom descriptor, with each tab corresponding to a different set of predictions in non-water solvents.
4. **minnesota_error_atom_water** - Summary of error for atom descriptor, with each tab corresponding to a different set of predictions in water.
5. **minnesota_error_bond** - Summary of error for bond descriptor, with each tab corresponding to a different set of predictions.
6. **minnesota_error_bond_nonwater** - Summary of error for bond descriptor, with each tab corresponding to a different set of predictions in non-water solvents.
7. **minnesota_error_bond_water** - Summary of error for bond descriptor, with each tab corresponding to a different set of predictions in water.
8. **minnesota_error_hba1** - Summary of error for HBA1 (hydrogen bond acceptor) descriptor, with each tab corresponding to a different set of predictions.
9. **minnesota_error_hba1_nonwater** - Summary of error for HBA1 (hydrogen bond acceptor) descriptor, with each tab corresponding to a different set of predictions in non-water solvents.
10. **minnesota_error_hba1_water** - Summary of error for HBA1 (hydrogen bond acceptor) descriptor, with each tab corresponding to a different set of predictions in water.
11. **minnesota_error_hba2** - Summary of error for HBA2 (hydrogen bond acceptor) descriptor, with each tab corresponding to a different set of predictions.
12. **minnesota_error_hba2_nonwater** - Summary of error for HBA2 (hydrogen bond acceptor) descriptor, with each tab corresponding to a different set of predictions in non-water solvents.
13. **minnesota_error_hba2_water** - Summary of error for HBA2 (hydrogen bond acceptor) descriptor, with each tab corresponding to a different set of predictions in water.
14. **minnesota_error_hbd** - Summary of error for HBD (hydrogen bond donor) descriptor, with each tab corresponding to a different set of predictions.
15. **minnesota_error_hbd_nonwater** - Summary of error for HBD (hydrogen bond donor) descriptor, with each tab corresponding to a different set of predictions in non-water solvents.
16. **minnesota_error_hbd_water** - Summary of error for HBD (hydrogen bond donor) descriptor, with each tab corresponding to a different set of predictions in water.
17. **minnesota_error_rotors** - Summary of error for rotors (torsional angles) descriptor, with each tab corresponding to a different set of predictions.
18. **minnesota_error_rotors_nonwater** - Summary of error for rotors (torsional angles) descriptor, with each tab corresponding to a different set of predictions in non-water solvents.
19. **minnesota_error_rotors_water** - Summary of error for rotors (torsional angles) descriptor, with each tab corresponding to a different set of predictions in water.
20. **minnesota_error_solvent** - Summary of error broken down by solvent, with each tab corresponding to a different set of predictions.
21. **minnesota_error_solvent_class** - Summary of error broken down by uESE solvent class, with each tab corresponding to a different set of predictions. The uESE solvent classes are: A (water), B (polar protic), C (polar aprotic), and D (nonpolar).

## dGSolvDB1

The folder contains all of the results for the dGsolvDB1 database in the 4 spreadsheets below. Results are provided for set 1 predictions.

1. **dGsolvDB1_all_data_solvation_fe** - The tab “dGsolvDB1_uESE” contains the results from the present study, and “references” contains the original references from the dGsolvDB1 database.
2. **dGsolvDB_error** - A summary of the error, where the first six tabs corresponds to a separate descriptor as labeled, the seventh tab (“solvent class”) corresponds to the solvent class, the eighth tab (“solvent”) is a breakdown by solvent, and the ninth tab (“solute”) is a breakdown by solute.
3. **dGsolvDB_error_nonwater** - A summary of the error, where the six tabs correspond to a separate descriptor as labeled, in non-water solvents.
4. **dGsolvDB_error_water** - A summary of the error, where the six tabs correspond to a separate descriptor as labeled, in water.
